# Clathrin mediated endocytosis targeting chimera for targeted membrane proteins degradation and enhance CAR-T cell anti-tumor therapy

**DOI:** 10.7150/thno.114005

**Published:** 2026-01-01

**Authors:** Yishuang Li, Zhihong Zhou, Yonghui Lv, Haoneng Hu, Ming Chen, Kunjian Lei, Chuandong Gong, Junzhe Liu, Lufei Yang, Linzhen Huang, Wenji Liu, Jinying Li, Minhua Ye, Xingen Zhu, Kai Huang

**Affiliations:** 1 Department of Neurosurgery, The 2nd affiliated hospital, Jiangxi Medical College, Nanchang University, Nanchang, China.; 2 Jiangxi Province Key Laboratory of Neurological Diseases, Nanchang, China.; 3 JXHC key Laboratory of Neurological medicine, Nanchang, China.; 4 Institute of Neuroscience, Nanchang University, Nanchang, China.; 5 The MOE Basic Research and Innovation Center for the Targeted Therapeutics of Solid Tumors, The 2nd Affiliated hospital, Jiangxi Medical College, Nanchang University, Nanchang, China.; 6 Second Clinical Medical School, Jiangxi Medical College, Nanchang University, Nanchang, China.; 7 School of Pharmacy, Jiangxi Medical College, Nanchang University, Nanchang, China.

**Keywords:** Targeted protein degradation, CleTAC, Cancer therapy, Endocytosis degradation, CAR-T cell therapy

## Abstract

**Rationale:** T cell exhaustion, mediated by the expression of inhibitory receptor proteins, significantly reduces their anti-tumor efficacy. Therefore, strategies aimed at degrading membrane proteins have emerged as promising approaches for enhancing the therapeutic effectiveness of Chimeric Antigen Receptor (CAR) T cells and improving cancer treatment outcomes.

**Methods:** In this study, we developed a Clathrin-Mediated Endocytosis Targeting Chimera (CleTAC), an innovative platform designed to facilitate membrane protein degradation via the clathrin-mediated endocytosis pathway. CleTAC employs the YVKM motif to interact with the AP2 complex, driving the internalization of targeted membrane proteins (proteins of interest, POI) along with associated cell membrane components. These internalized complexes are subsequently trafficked to lysosomes for degradation. We conducted multiple validations using flow cytometry, Western blotting, and *in vitro* and *in vivo* experiments to verify its degradation efficiency. Additionally, we integrated CleTAC targeting CTLA4 membrane proteins into a CAR construct and evaluated its impact on CAR-T cell functionality and tumor suppressive efficacy using both cellular assays and animal tumor models.

**Results:** We demonstrated that CleTAC effectively and specifically mediates the degradation of EGFP and CTLA4 proteins on the cell surface. When incorporated into a CAR construct, CleTAC targeting CTLA4 enhanced CAR-T cell anti-tumor activity, as evidenced by improved functional assays and tumor suppression in animal models. These findings establish CleTAC as a versatile and effective tool for modulating membrane protein levels and enhance anti-tumor efficacy in CAR-T cells.

**Conclusions:** Our results highlight CleTAC as a promising therapeutic tool with substantial clinical potential to significantly enhance CAR-T cell therapy and advance current oncology treatment strategies.

## Introduction

CAR-T cell immunotherapy represents an innovative and increasingly utilized therapeutic strategy in cancer treatment. This approach involves genetically modifying patient-derived T cells *ex vivo* to specifically target tumor-associated antigens, enabling potent anti-cancer activity 1. Despite significant successes, CAR-T cell therapy continues to face notable limitations, such as antigen escape, limited persistence, and T cell exhaustion 2-4. T cell exhaustion, primarily driven by the expression of inhibitory receptor proteins, considerably impairs the sustained anti-tumor functions of engineered T cells 5. Prominent inhibitory receptors contributing to this exhaustion include Programmed Cell Death Protein 1 (PD-1), T cell immunoglobulin mucin domain-3 (TIM-3), Cytotoxic T Lymphocyte-Associated Antigen 4 (CTLA4), and T cell immunoglobulin and ITIM domain (TIGIT) 2,6-10.

CTLA4, a transmembrane glycoprotein expressed on activated T cells, reduce immune responses by competing with CD28 for binding to B7 molecules on antigen-presenting cells 11,12. Therapeutic blockade of the B7-CTLA-4 axis—via monoclonal antibodies (e.g., ipilimumab) or genetic ablation (e.g., CRISPR-Cas9) has demonstrated substantial efficacy in multiple malignancies 13-18. However, CRISPR-based gene editing could result in permanent genomic alterations with safety concerns, while antibody-based therapies may trigger hyper-progressive diseases or autoimmune reactions 19,20.

Clathrin-mediated endocytosis (CME) is a well-characterized cellular process for the internalization of membrane proteins through vesicle formation 21,22. The AP2 adaptor complex, recognizing specific cytoplasmic motifs such as the YXXΦ motif where Φ is a hydrophobic residue, facilitating vesicle formation and internalization of target proteins 22-24. This process plays a vital role in regulating membrane protein composition, the immune system, and maintaining internal environment homeostasis 25.

In this study, we propose integrating CME-based targeted degradation into CAR-T cell therapy to optimize anti-tumour efficacy by reducing CTLA4 expression. Our engineered CleTAC utilizes the YVKM motif, enhancing CTLA4 internalization and subsequent degradation. We introduce CleTAC and validate its efficacy through cellular and animal tumour model studies, demonstrating that engineered CAR-T cells with reduced membrane CTLA4 exhibit improved anti-tumour performance. This innovative approach highlights CleTAC's therapeutic potential in advancing CAR-T immunotherapy and broadening applications of targeted protein degradation in oncology treatment strategies.

## Results

### Engineered YVKM-fusion degraders lead to efficient degradation of EGFP

CME is a highly conserved cellular process responsible for the selective internalization of transmembrane receptors and signaling molecules. This mechanism operates through adaptor protein complexes, notably the AP2 complex, which recognizes cytosolic sorting motifs of the form YXXΦ on cargo proteins, thereby promoting their clustering within clathrin-coated pits 24,26. Among the YXXΦ families, motifs such as YVKM in CTLA4, YIPL in hepatitis C virus (HCV), YRRF in m04/gp34, YQRL in TGN38, and YERV in EREG have been well characterised as basolateral sorting signals. 27-31. Comparative quantitative analyses revealed that the YVKM motif exhibits the most rapid degradation kinetics, identifying it as a prime candidate for further functional exploration (Figures S1A-B).

The tyrosine residue (Y) within the YVKM is crucial for AP2 binding and efficient internalization; substitution of this residue with phenylalanine (Y→F) markedly reduces AP2 affinity and impairs downstream degradation 27,32. To exploit this insight, we fused YVKM to a GFP-specific nanobody (VHH), generating CleTAC-v1 constructs (VHH-YVKM) that bind EGFP intracellularly. Flow cytometry-based analyses conducted in 293T cells transfected with CleTAC-v1 constructs demonstrated significant EGFP degradation (Figures S1A-D).

Current membrane protein degradation strategies predominantly employ antibody-based extracellular recognition, typically through single-chain variable fragments (scFv) 33,34. To test this approach in our system, we generated lentiviral vectors encoding extracellular scFv-based CleTAC constructs and transduced 293T cells. Although this strategy achieved robust EGFP degradation (Figures S1C-D), microscopic examination revealed pronounced cell aggregation (Figure S1E). Given the precedent established by CAR-T fratricide phenomena 35, we speculated that extracellular binding sequences might similarly induce fratricide in T cells. Further studies indeed showed significant fratricide in T cells transfected with extracellular EGFP-targeting constructs (Figures S2). To avoid these complications, we therefore relocated the targeting sequence to the intracellular compartment, preserving degradation efficacy while eliminating unwanted cell-cell cytotoxicity.

**Figure 1:**
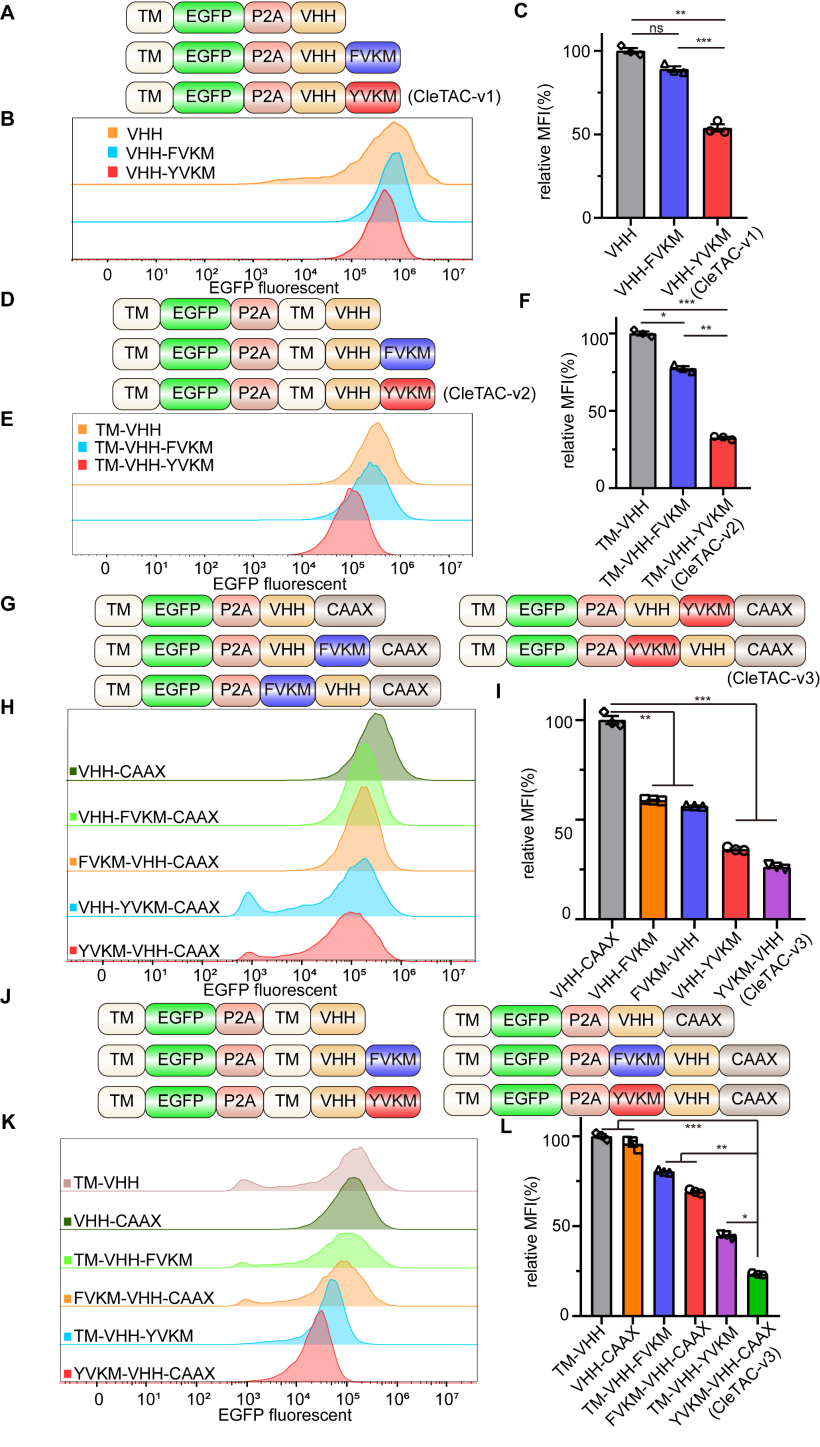
** Development of CleTAC degraders for targeted EGFP degradation through protein engineering.** (A) Schematic illustrations of the first generation of CleTAC degraders (VHH-YVKM) which VHH represents GFP-nanobody. (B) Distribution of EGFP fluorescent cells in 293T cells stably expressing VHH-YVKM. (C) The relative mean fluorescence intensity (MFI) exhibited by VHH, VHH-FVKM, or VHH-YVKM after intracellular expression was displayed. (D) Schematic illustrations of the second generation of CleTAC degraders (TM-VHH-YVKM) highlighting the transmembrane (TM) domain from PGFRB protein. (E) Distribution of EGFP fluorescent cells in 293T cells stably expressing TM-VHH-YVKM. (F) The relative MFI exhibited by TM-VHH, TM-VHH-FVKM, or TM-VHH-YVKM after intracellular expression was displayed. (G) Schematic illustrations of the third generation of CleTAC degraders (YVKM-VHH-CAAX) highlighting the farnesylation via CAAX motif. (H) Distribution of EGFP fluorescent cells in 293T cells stably expressing VHH-YVKM-CAAX and YVKM-VHH-CAAX. (I) The MFI exhibited by VHH-CAAX, VHH-YVKM-CAAX, or YVKM-VHH-CAAX after intracellular expression was displayed. (J-L) The comparison between TM-VHH-YVKM and YVKM-VHH-CAAX in which the control groups are TM-VHH and VHH-CAAX (J), histogram analyses of EGFP fluorescent (K), and relative MFI (L).

In order to validate our approach, we generated two control constructs: a VHH-only control and an FVKM mutant (Y→F substitution) (Figure 1A). Luminescence from the VHH group were normalised to 100%. Under these conditions, YVKM-VHH CleTACs exhibited approximately 50% EGFP degradation (Figures 1B-C), whereas the FVKM mutant effected less than 15% degradation. These results demonstrates that the tyrosine residue within the YVKM motif is essential for efficient CME-meditated degradation. Although degradation efficiency was moderate, the successful targeted EGFP degradation using CleTACs highlights its potential for targeted membrane protein degradation, underscoring their promising clinical implications for enhancing CAR-T therapeutic strategies.

### Enhancing CleTAC efficiency through transmembrane anchoring and farnesylation strategies

Previous research has highlighted the close relationship between clathrin-mediated endocytosis, membrane proteins, and membrane invagination and bud formation 22. To improve efficacy of binding and degradation, we optimized the CleTAC design by incorporating a transmembrane (TM) sequence from PDGFRB protein upstream of the GFP-nanobody (VHH) (Figure 1D). This TM domain anchors EGFP and VHH to the cell membrane, indirectly enhancing their interaction and thus promoting more efficient YVKM-mediated EGFP degradation. Indeed second-generation CleTAC-v2 constructs (TM-VHH-YVKM) containing the TM sequence resulted in at least 60% EGFP degradation efficiency (Figures 1E-F).

We also explored protein farnesylation as an alternative membrane-anchoring strategy for YVKM. Farnesylation is a form of prenylation that installs a hydrophobic farnesyl group onto proteins bearing a C-terminal CAAX motif, (C= cysteine; AA = two aliphatic residues; X = variable residue) 36-39. To verify the confirmation of increased membrane targeting by TM and CAAX, we used DIL orange red cell membrane fluorescent dye and observed its overlap with EGFP green fluorescence (Figure S2E). We then appended a CAAX motif to the C-terminus of our first-generation CleTAC constructs to produce third-generation variants (Figures 1G-I). Remarkably, these CAAX-modified CleTAC-v3 (YVKM-VHH-CAAX) achieved over 70% EGFP degradation (Figures 1J-L), demonstrating that combining YVKM with CAAX-mediated membrane anchoring significantly enhances targeted protein degradation.

### Enhancing targeted protein degradation through linker length modulation and YVKM motif multiplicity in CleTACs

**Figure 2:**
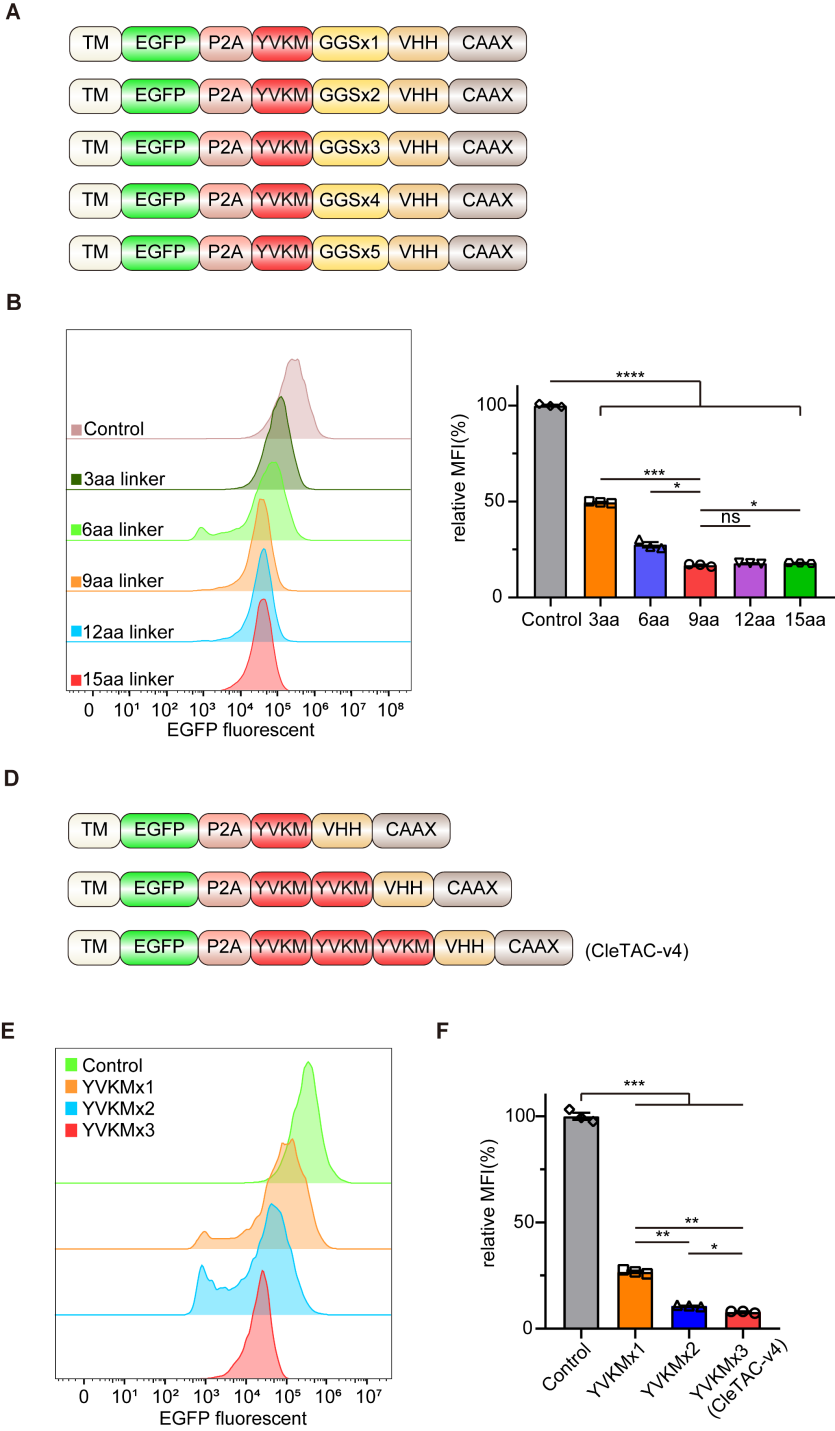
** Development of CleTAC degraders for targeted EGFP degradation via adjusting linker length and multivalent structure.** (A) Schematic illustrations of the 4th generation of CleTAC degraders which highlights different length of linkers. (B) Distribution of EGFP fluorescent cells in 293T cells stably expressing different linkers 4th generation of CleTAC. (C) The relative MFI exhibited by 2-10aa CleTACs after intracellular expression was displayed. (D) Schematic illustrations of the 5th generation of CleTAC degraders which highlights multivalency constructions of YVKM motifs. (E) Distribution of EGFP fluorescent cells in 293T cells stably expressing different multivalency constructions of 5th generation of CleTAC. (F) The relative MFI exhibited by 1-3 repetitive motifs after intracellular expression was displayed.

Flexible glycine (Gly)- serine (Ser) linkers are known to enhance fusion-protein solubility and flexibility thereby facilitating movement between protein domains 40,41. Previous studies have shown that the length, type, and sequence of linkers can significantly influence the biological function of fusion proteins 40,42. Another strategy to enhance degradation efficiency involves multivalency, which increases protein binding affinity and specificity 43,44. We therefore designed fourth-generation CleTACs by varying linker lengths from 3 to 15 amino acids (aa) and incorporating multiple YVKM motifs (Figures 2A and 2D). Consistent with previous findings 44, degradation efficiency did not uniformly increase with linker length but plateaued at a 9-aa linker (Figures 2B-C). Among these designs, CleTACs containing a 6-aa linker with triple YVKM motifs achieved over 90% targeted degradation of EGFP (Figures 2E-F). These findings clearly indicate that a suitable linker length, coupled with repeated YVKM motifs in CleTAC-v4 constructs, significantly enhances targeted membrane protein degradation efficiency.

In addition, proteomic analysis was performed to assess the off-target effects of CleTAC. The proteomic analysis results indicated that CleTAC-mediated degradation of exogenous EGFP had minimal impact on the levels of other membrane proteins, suggesting favorable off-target profiles (Figure S4F). And the expression of other membrane proteins was not significantly reduced.

### Elucidating the role of AP2 adaptor complex in CleTAC-driven clathrin-dependent protein degradation

**Figure 3:**
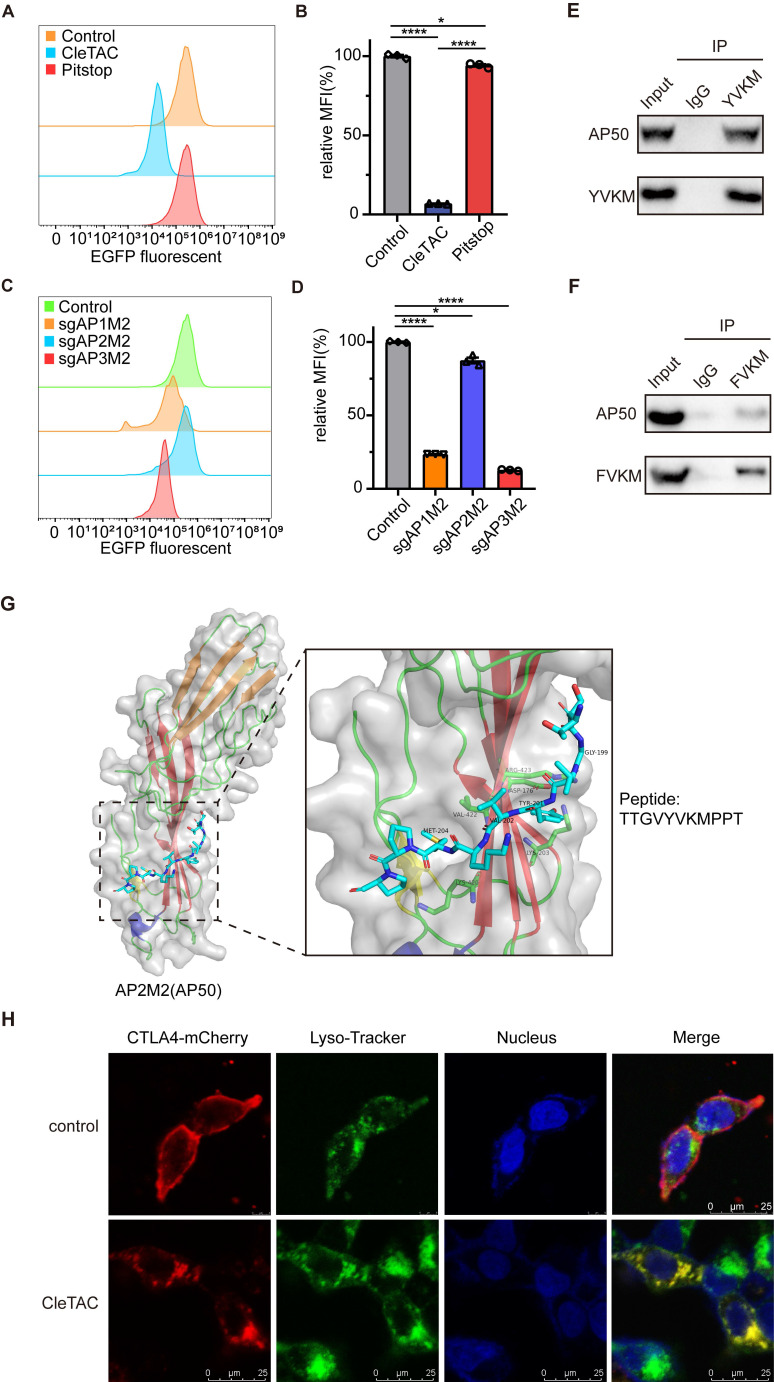
** Clathrin-mediated endocytosis plays a key role in EGFP targeted degradation of CleTAC.** (A) Distribution of EGFP fluorescent cells in 293T cells stably expressing CleTAC with pitstop. (B) The effect of pitstop inhibitor on the degradation efficiency of CleTAC measured by flow cytometry. (C) Distribution of EGFP fluorescent cells in CleTAC-293T cells which knockout AP1M1, AP2M1 and AP3M1 by CRISPR/Cas9. (D) The relative MFI exhibited by sgAP1M1, sgAP2M1 and sgAP3M1 CleTAC-293T cells after intracellular expression was displayed. (E and F) Co-immunoprecipitation (co-IP) of AP50 from 293T cells after transfection of plasmids that encode AP50 and CleTAC degraders (YVKM in E and FVKM in F). AP50 was immunoprecipitated via the HA-tag; AP50-bound protein complexes were then captured from the lysates and blotted for the HA-tag (AP50) and FLAG-tag (CleTAC degrader). (G) Structure of AP2M2(AP50) protein and poseview of amino acid interactions of YVKM peptide with AP50. PDB ID: 1H6E. (H) Representative images for the cellular localization of CleTAC degrader and Lyso-Tracker in live 293T cells. Scale bar: 20μm.

To confirm that CleTACs mediate EGFP degradation specifically occurs via the clathrin-mediated endocytosis, we employed Pitstop2, a specific inhibitor that binds the N-terminal domain of clathrin and disrupts its interaction with adaptor proteins, thus interfering with receptor-mediated internalization 45,46^.^ Flow cytometry analyses demonstrated that Pitstop2 significantly inhibited CleTAC-induced EGFP degradation (Figures 3A-B), confirming the dependence of our system on the clathrin-mediated pathway.

The clathrin-mediated endocytosis pathway involves three primary adaptor complexes: AP1, AP2, and AP3. To investigate their individual roles, we employed CRISPR/Cas9 gene editing to generate AP1M1-, AP2M1-, and AP3M1-knockout 293T cell lines, verifying successful knockout through immunoblot analysis (Figures S3A-C). Previous research established that the interaction between the YVKM motif and AP50, a medium-chain subunit of the AP2 complex, is crucial for clathrin-mediated endocytosis 27,47. Consistent with these findings, knockout of AP2M1 substantially reduced EGFP degradation mediated by CleTAC, whereas knockout of AP1M1 and AP3M1 showed minimal effects on degradation efficiency (Figures 3C-D).

The interaction between AP50 and the YVKM motif was confirmed by co-immunoprecipitation (co-IP) assays. 293T cells expressing either the wild-type or FVKM mutant CleTAC constructs were lysed, and tagged AP50 was precipitated; robust co-precipitation of the wild-type CleTAC, but not the FVKM variant, demonstrated motif-dependent binding (Figures 3E-F and S3D). Additionally, structural analysis of AP50 via X-ray crystallography revealed specific hydrogen bonding interactions between AP50 and the CTLA4 peptide fragment, primarily localized at the YVKM motif (Figure 3G).

To further verify the subcellular localization and trafficking of CleTAC, we performed immunefluorescence assays. In the absence of YVKM motif, fluorescent signals predominantly localized to the cell membrane, whereas YVKM-containing constructs were internalized and colocalized with lysosomal markers. In the presence of Pitstop2, internalization was inhibited, resulting in fluorescence remaining predominantly at the cell surface, similar to the control conditions (Figure 3H).

RT-qPCR analysis showed that CleTAC treatment did not affect the mRNA levels of target proteins, confirming that degradation occurs post-transcriptionally (Figures S4A-C). Together, these data demonstrate that CleTAC mediates target clearance via a YVKM-dependent, clathrin-mediated endocytosis mechanism.

### Mitigating CAR-mediated trogocytosis and enhancing CAR-T cell efficacy through CleTAC-mediated HER2 and CTLA4 degradation

During T cell-related experiments, we observed an unexpected increase in the surface expression of HER2 antigen on CAR-T cells following stimulation with 251 tumor cells (Figures S5A-C). To trace the origin of these HER2 antigens, we evaluated HER2 surface expression on the remaining 251 cells. Quantitative analysis revealed a significant decrease in HER2 surface levels on 251 cells post-CAR-T treatment compared to untreated controls (Figures S5D-F). These observations are consistent with CAR-mediated trogocytosis (CMT), whereby activated T cells extract tumor antigens, leading to decreased antigen density on malignant cells and the appearance of antigen-positive CAR-T cells 48-50. CMT can facilitate the emergence of antigen-negative escape variants and has been shown to impair CAR-T efficacy via fratricidal T-cell killing and the induction of T cell exhaustion 35,51,52.

Given the negative impact of CMT on CAR-T cells, we employed RFdiffusion and ProteinMPNN methodologies to design three novel proteins capable of binding to the HER2 inner membrane region (Figures S6A-B) 53,54. These designed proteins underwent rigorous biochemical validation via quantitative co-immunoprecipitation (co-IP) assays, confirming their high binding affinity (Figure S6C). Subsequently, we replaced the VHH sequence in the CleTAC plasmid with the validated HER2-binding protein, constructing the HER2-CleTAC plasmid, which was then introduced into T cells through electroporation. CleTAC-mediated HER2 removal significantly reduced CMT occurrence, thereby preventing fratricidal T cell death and exhaustion, ultimately enhancing CAR-T cell tumoricidal efficacy (Figures S5G-K).

**Figure 4:**
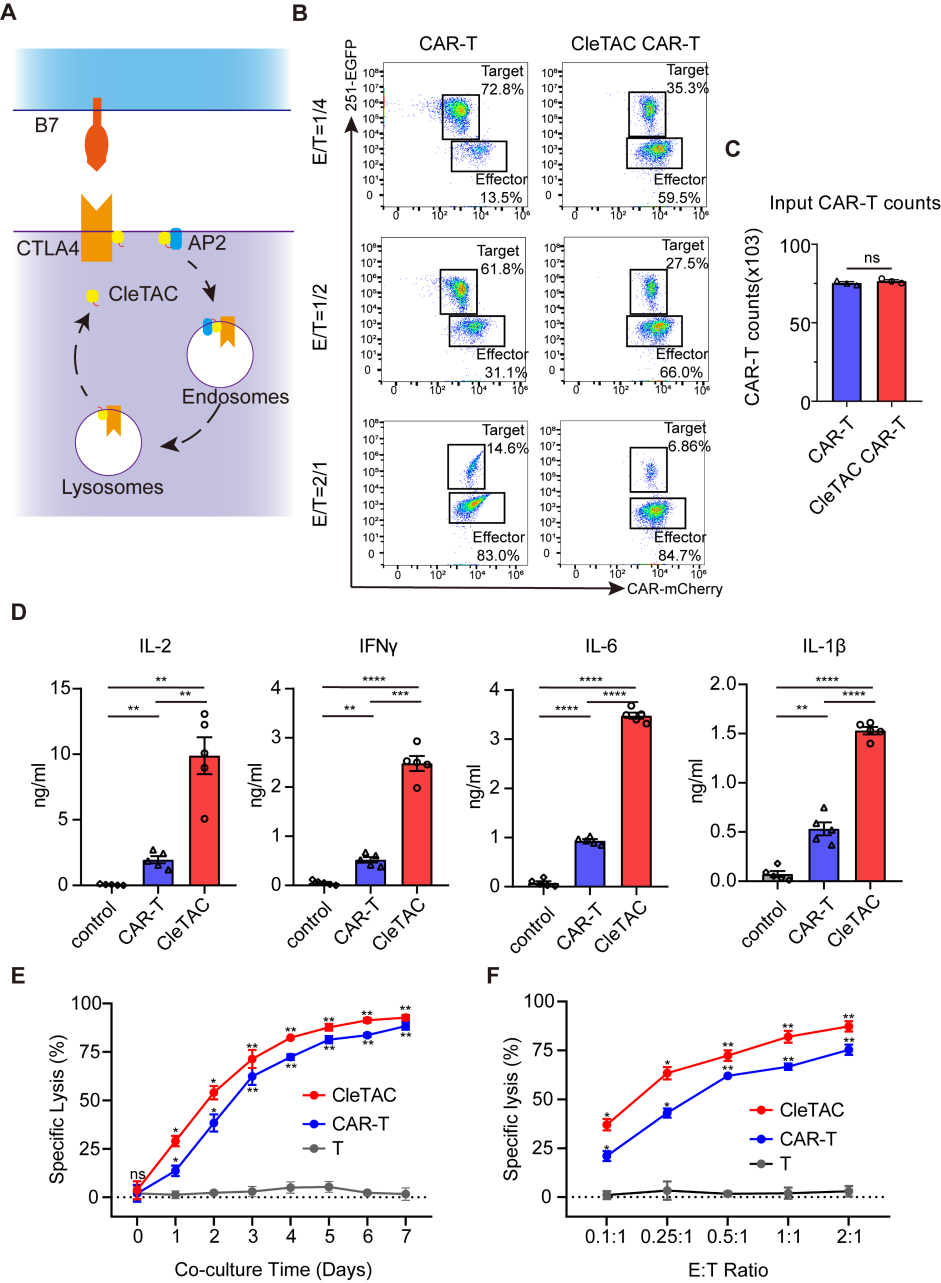
** The CleTAC-mediated CTLA4 degradation can enhance CAR-T cell efficacy.** (A) Schematic diagram of the principle of CTLA4 targeted degradation by CleTAC. (B) Representative flow cytometry results showing CAR-T and CleTAC CAR-T killing capability under U251 stimulation. (C) Quantification of input CAR-T cell counts without U251 stimulation showing relatively equal amount of input CAR-T cells, n = 3, ns=p>0.05. (D) Quantification of intracellular IL-2 (n = 5), IFN-γ (n = 5) IL-6 (n = 5) and IL-1β (n = 5) expression. For all bar plots, data are shown as mean ± SEM. (E and F) Cytotoxic activity of CAR-T or CleTAC CAR-T against U251-EGFP at different co-culture time and E:T ratios (n = 3).

To further demonstrate CleTAC efficacy, we replaced EGFP with the immune checkpoint protein CTLA4 while retaining the N-terminal YVKM motif and C-terminal CAAX sequence. Using the interaction between CTLA4 and serine/threonine phosphatase 2A (PP2A) 55,56, we developed a CleTAC variant in which the VHH domain was replaced with a PP2A-binding module to facilitate interaction between CTLA4 and the YVKM motifs, resulting in a novel CleTAC construct for CTLA4 degradation (Figure 4A). We then compared CTLA-4 levels in CleTAC-treated and control cells by Western blotting and quantitative PCR (Figures S4D-E). CleTAC treatment resulted in a pronounced decrease in CTLA-4 protein, whereas CTLA-4 mRNA remained unchanged, confirming that CleTAC mediates post-translational degradation of its membrane protein target without affecting transcription. The concentration- and time-dependent degradation of the protein of interest (POI) by CleTAC was systematically evaluated via WB. Experimental groups were established with varying concentrations (1-4 μg) and durations (6 h, 12 h, 24 h, and 36 h). The results demonstrated a clear dose-response relationship between CleTAC concentration and POI degradation levels, confirming that the observed effects are both reproducible and quantitatively dependent on the concentration and duration of CleTAC (Figures S4G-H). Based on the optimized concentration and duration selected from these experiments, we consistently used 24 h and 3 μg of CleTAC in subsequent transfection experiments.

To investigate whether PP2A would affect downstream signaling pathways, we compared the expression levels of ERK1/2 and phosphorylated ERK1/2 in T cells transfected with the CleTAC plasmid system containing the PP2A scaffold subunit (PP2AA) sequence and in the control group. No significant differences in either total or phophorylated ERK1/2 levels were detected between the two groups (Figure S7A).

In addition, CD28-B7 is a well-known T cell activation signaling pathway that is essential for the co-stimulation and activation of T cells We also evaluated whether CleTAC-mediated CTLA-4 degradation affects the CD28-B7 co-stimulatory pathway by measuring AKT and phosphorylated AKT levels, as well as the secretion of granzyme B, IL-2, and IFN-γ in CleTAC-treated versus control T cells (Figures S7B-C). Contrary to concerns of pathway disruption, CTLA-4 removal enhanced CD28-B7 signaling and T-cell activation. Consistently, a CCK-8 proliferation assay demonstrated increased CAR-T cell activity following CTLA-4 degradation (Figure S7D), further supporting our conclusion that CleTAC augments, rather than impairs, co-stimulatory signaling.

The CAR-T cells used in this study were generated from peripheral blood mononuclear cell (PBMC) obtained from healthy donors, with the 251-glioma cell line serving as the target. We co-cultured both standard CAR-T and CAR-CleTAC cells with 251-GFP cells at multiple effector-to-target (E:T) ratios and quantified cytotoxicity by measuring luminescence relative to tumor-only control wells (set to 100%). Although both cell types were seeded at equal effector numbers, CAR-CleTAC cells exhibited significantly enhanced antitumor activity, as indicated by a greater decrease in viable 251-GFP cells compared with conventional CAR-T cells (Figures 4B-C).

Furthermore, CleTACs-engineered CAR-T cells secreted significantly higher levels of IL-2, IFN-γ, IL-6, and IL-1β compared to unstimulated controls (Figure 4D). To evaluate sustained cytotoxicity, CleTAC and conventional CAR-T cells were co-cultured with U251-luciferase target cells at various E:T ratios for seven days. Longitudinal luciferase assays demonstrated that CleTAC CAR-T cells consistently achieved greater tumour cell elimination than conventional CAR-T cells across all tested ratios (Figures 4E-F). Collectively, flow cytometry and luciferase-based assays confirm that CleTAC-mediated CTLA-4 protein degradation significantly enhances the therapeutic efficacy of CAR-T cells.

### Mitigating T cell exhaustion in CAR-T therapy through CleTAC-mediated CTLA4 degradation

**Figure 5:**
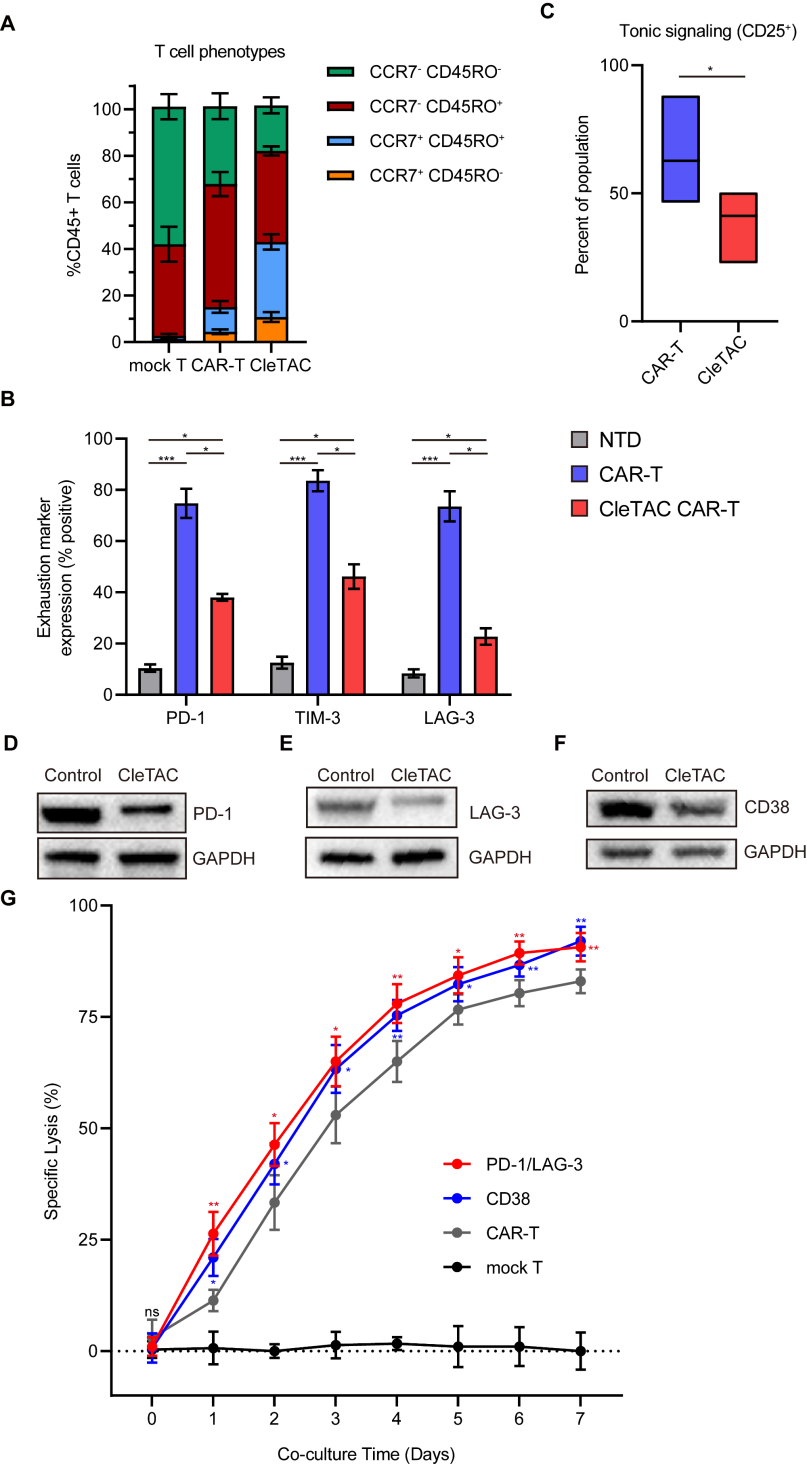
** The CleTAC CAR-T cells show a more stem-like phenotype and reduced exhaustion.** (A) Quantification (n = 3) showing memory phenotype of different CAR-T cells. Memory phenotype populations are defined as: naive-like (CCR7^+^ CD45RO^-^), central memory (CM; CCR7^+^ CD45RO^+^), effector memory (EM; CCR7^-^ CD45RO^+^), effector (effector memory cells re-expressing CD45RA [EMRA]; CCR7^-^ CD45RO^-^). Error bars indicate mean ± SEM in each memory sub-population. (B) Expression of exhaustion markers (PD1, LAG3, or TIM3) by non-transduced T cells, CAR-T and CleTAC CAR-T cell (n = 3). Error bars indicate mean ± SEM. (C) Tonic signaling in CAR-T versus CleTAC CAR-T cells, measured by percent CD25^+^ cells (P < 0.05, n = 3). Boxes represent min to max with median center line. (D-F) Western blot analysis of PD-1 (D)/ LAG-3 (E) and CD38 (F) levels in CleTAC-mediated degradation CAR-T cells. (G) Cytotoxic activity of PD-1/LAG-3 and CD38 CleTAC CAR-T against U251-EGFP at different co-culture time (n = 3).

T cell exhaustion poses a significant barrier to effective CAR-T therapy in solid tumors by undermining long-term antitumor function. Recent work indicates that tonic signaling from constitutively active CAR constructs increases exhaustion, reducing T cell survival and proliferation *in vivo* 57,58. To address this limitation, we used flow cytometry to compare differentiation profiles among engineered T cell populations. CleTAC CAR-T cell populations retained a higher frequency of naïve-like T cells (characterized by CCR7⁺CD45RA⁺ expression), a subset associated with enhanced *in vivo* persistence and superior antitumor efficacy 59,60, compared to constitutively active CAR-T cells (Figure 5A).

To further characterize T cell exhaustion, we examined the expression levels of critical immune checkpoint markers, including PD-1, TIM-3, and Lymphocyte Activation Gene 3 (LAG-3) (Figure 5B). Constitutive CAR-T cells exhibited significantly elevated expression of these exhaustion markers relative to both non-transduced and CleTAC CAR-T cells. Notably, PD-1 and TIM-3 expression was significantly elevated in constitutive CAR-T cells compared to non-transduced controls (P < 0.05), whereas CleTAC CAR-T cells-maintained exhaustion marker expression comparable to non-transduced controls, with no significant differences detected.

Since tonic signaling from constitutive CAR expression is a key driver of T cell exhaustion 57,58, we hypothesized that CleTAC CAR-T cells, which theoretically experience reduced tonic signaling, would demonstrate decreased exhaustion and enhanced maintenance of a stem-like phenotype. Indeed, CleTAC CAR-T cells exhibited diminished tonic signaling relative to constitutively active CAR-T cells, as evidenced by lower CD25 expression levels under antigen-free conditions (Figure 5C). The expression level of CD25 is closely associated with the strength of tonic signaling, the autoreactivity of T cells, and their functional state 61,62. High tonic signaling is typically accompanied by elevated CD25 expression. Excessive tonic signaling (e.g., signal amplification caused by spontaneous aggregation of CAR molecules) leads to increased CD25 expression, which in turn accelerates senescence and inhibits the functionality of CAR-T cells. Collectively, our findings suggest that targeted degradation of CTLA4 protein using the CleTAC system effectively mitigates tonic signaling, consequently preserving T cell differentiation potential and reducing exhaustion.

To further demonstrate the flexibility of the CleTAC platform, we applied it to dual and single targeting of inhibitory receptors. Based on reports that CD38 marks exhausted T cells and that its removal may boost CAR-T efficacy 63. we generated constructs for CD38 degradation alongside those targeting both PD-1 and LAG-3. We validated receptor knockdown by Western blot and assessed functional impact using luciferase-based cytotoxicity assays. As shown in Figures 5D-G, simultaneous PD-1/LAG-3 knockdown and CD38 degradation each produced a significant enhancement of CAR-T cell cytotoxic activity.

### Enhanced *in vivo* anti-tumor efficacy of CleTAC CAR-T cells in solid tumor models

**Figure 6:**
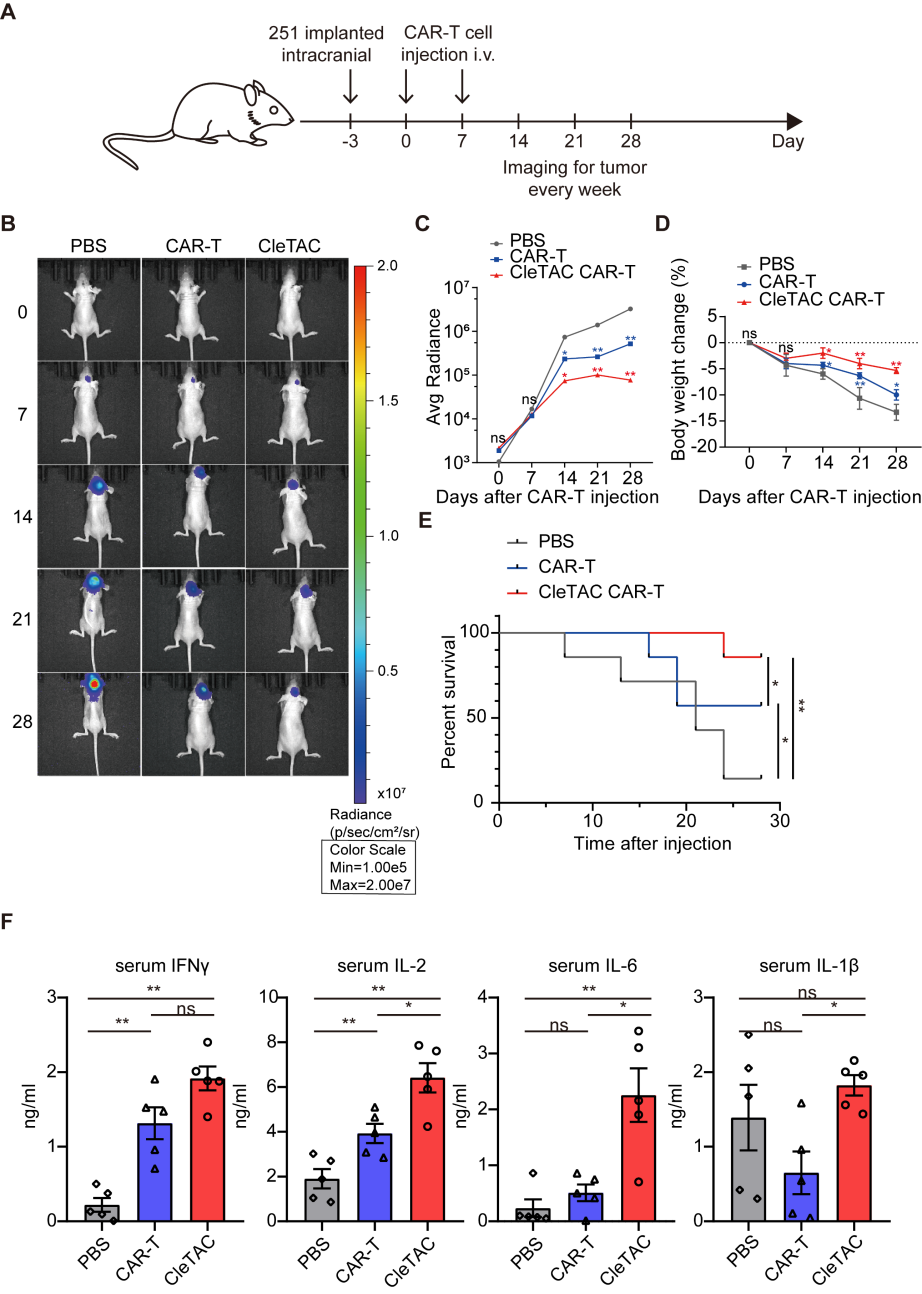
** CTLA4 degradation via CleTACs enhances the anti-tumor efficacy of CAR-T cells *in vivo*.** (A) Flowchart for intracranial construction of the U251 model and subsequent treatment in female nude mice (4 weeks old). (B) Bioluminescence imaging of nude mice xenografted with luciferase-infected U251 tumor cells. From left to right shows PBS, CAR-T and CleTAC CAR-T treatment. (C, D) Average radiance of bioluminescence (C) and percentage of body weight change (D) for different treatment groups of nude mice. (E) Monitoring the survival status of mice after T cell injection. (F) Quantification of serum IL-2 (n = 5), IFN-γ (n = 5) IL-6 (n = 5) and IL-1β (n = 5) expression. For all bar plots, data are shown as mean ± SEM.

To investigate the *in vivo* antitumour efficacy of CleTAC CAR-T cells, we employed an intracranial HER2⁺ 251 glioma orthotopic model in immunodeficient nude mice. Animals were randomized into 3 groups receiving intravenous injections (I.V.) of PBS, conventional CAR-T cells, or 2×10^7^ CleTAC CAR-T cells on days 0 and 7 post tumor inoculation (Figure 6A). We monitored body weight and tracked intracranial tumor growth weekly using small animal imaging. While both CAR-T groups exhibited improved control of tumor growth than the PBS group (Figures 6B-C), CleTAC CAR-T group demonstrated significantly slower tumor progression compared with constitutive CAR-T cellss, highlighting its enhanced antitumor activity and clearance efficacy.

Tumour progression in PBS-treated mice led to marked weight loss due to cachexia. Conversely, CleTAC CAR-T cells treatment partially preserved bodyweight, whereas conventional CAR-T cell afforded less protection (Figure 6D). Survival analyses further revealed that mice receiving CleTAC CAR-T cells survived significantly longer than those in both the PBS and conventional CAR-T cell groups (Figure 6E). Consistent with their enhanced antitumor performance, serum cytokine measurements by enzyme-linked immunosorbent assays (ELISA) showed elevated secretion levels of IL-2, IFN-γ, IL-6, and IL-1β by CleTAC CAR-T cells, (Figure 6F). These results indicate that CleTAC CAR-T cells mediate effective tumor regression without inducing excessive toxicity compared to constitutive CAR-T cells.

To validate the consistency of these findings, we conducted additional experiments using a lower dosage of 2×10⁶ CleTAC CAR-T cells against U251 glioma. As shown in Figure S8, this lower dose group maintained potent anti-tumour activity.

We further evaluated CleTAC CAR-T cells in an additional solid tumor model—the HER2⁺ MDA-MB-231 breast carcinoma xenograft—and observed superior tumor control compared with constitutive CAR-T treatment (Figure S9), reinforcing the enhanced efficacy of the CleTAC approach.

**Figure 7:**
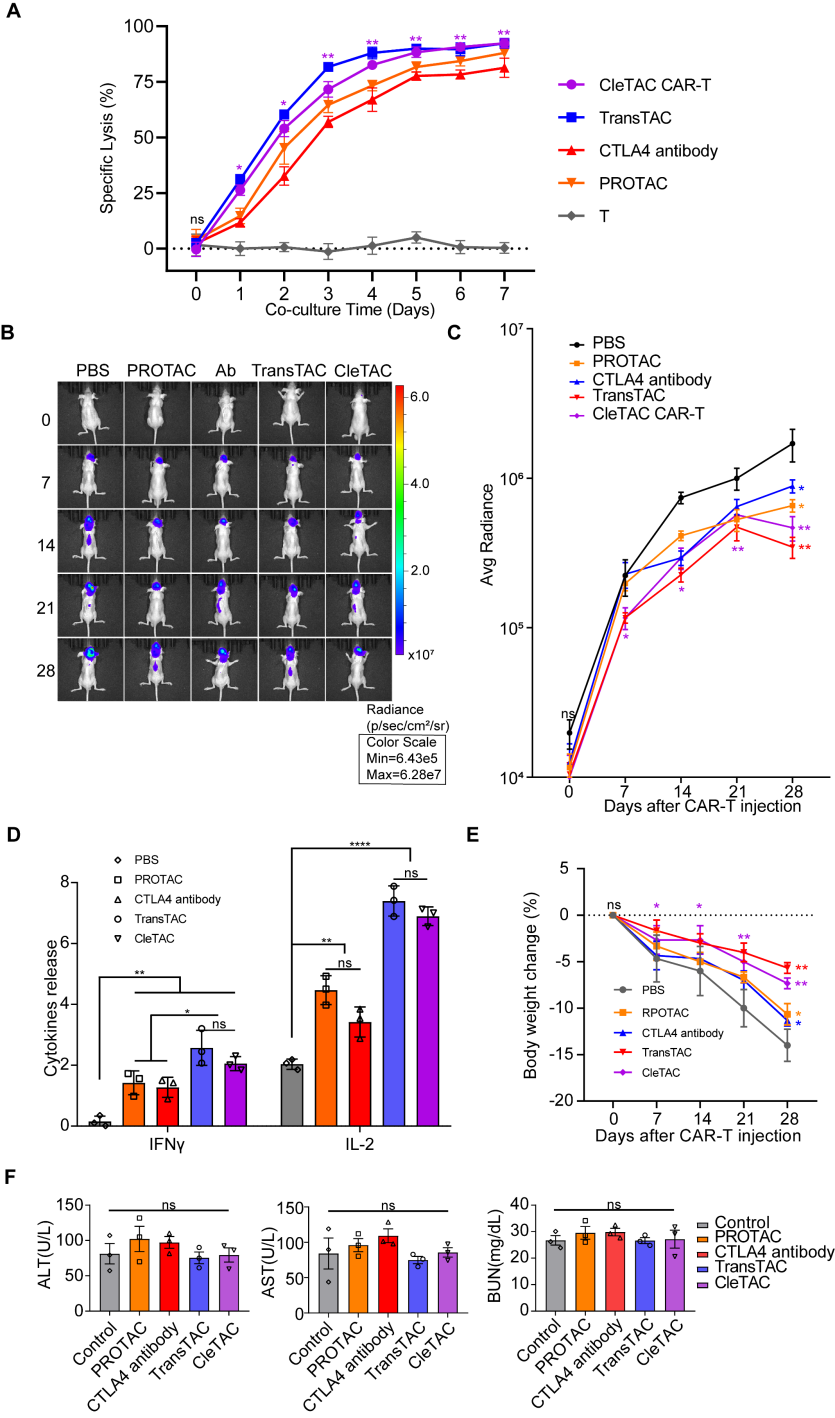
** Cletac-mediated CTLA4 degradation enhances CAR-T therapy compared with PROTAC, TransTAC, and CTLA4 antibodies.** (A) Cytotoxic activity of different CAR-T against U251-EGFP at different co-culture time (n = 3). (B) Bioluminescence imaging of nude mice xenografted with luciferase-infected U251 tumor cells. From left to right shows PBS, PROTAC, CTLA4 antibody with CAR-T, TransTAC and CleTAC CAR-T treatment (n = 3). (C) Average radiance of bioluminescence for different treatment groups of nude mice (n = 3). (D) Quantification of serum IFN-γ and IL-2 expression (n = 3). (E) Percentage of body weight change for different treatment groups of nude mice (n = 3). (F) Quantification of clinical biomarkers of toxicity AST, ALT and BUN for different treatment groups (n = 3). For all bar plots, data are shown as mean ± SEM.

To further verify the efficiency and practicability of the CleTAC approach, we condtucted direct comparsions with PROTAC, TransTAC and CAR-T combined with the anti-CTLA4 antibody ipilimumab. *In vitro*, T-cell cytotoxicity was measured by luciferase-based assays, and *in vivo* therapeutic performance was assessed in relevant animal models (Figures 7A-C). Safety was examined by monitoring clinical toxicity biomarkers (AST, ALT, and BUN) to confirm that CleTAC meets translational safety standards (Figure 7E). CleTAC demonstrated superior antitumor efficacy compared with both ipilimumab-based CAR-T therapy and PROTAC, while TransTAC demonstrated slightly superior performance over CleTAC.

## Discussion

Current techniques for targeted protein knockdown generally function by indirectly suppressing protein expression, most commonly through RNA interference or CRISPR-based gene knockout 64,65. Beyond the irreversible genomic modifications, these approaches are limited by inherent delays that can obstruct the timely suppression or knockout of target genes 66.

To overcome these challenges, we developed a CleTAC system for the precise degradation of proteins. We chose EGFP as an initial prototype because it enables real-time monitoring by flow cytometry and semi-quantitative analysis by Western blot, as well as rapid localization via fluorescence or confocal microscopy 67-69. Importantly, fusion of EGFP to target proteins generally does not alter their biological activity nor significantly affect cellular physiology 67,68. However, EGFP fluorescence could be quenched in acidic compartments such as lysosomes 70. To avoid it, we employed mCherry for immunofluorescence studies, providing stable fluorescence in acidic environments and ensuring accurate lysosomal localization 70.

Drawing on structural and functional insights into the YVKM and AP50 motifs—both essential for clathrin-mediated endocytosis— we engineered CleTAC constructs to degrade endogenous and exogenous proteins with high specificity. Each construct comprises three modules: an N-terminal YVKM motif that recruits the AP2 complex and initiates endocytosis; a central PP2A-binding domain, built on its conserved β-sheet scaffold, for direct target engagement; and a C-terminal CAAX box that directs farnesylation-dependent membrane anchoring 27,37,55,56. This modular architecture coordinates sequential target recognition, membrane localization, and lysosomal trafficking, providing precise spatiotemporal control over protein degradation.

We also showed that CleTAC effectively targets membrane proteins in 293T cells and modulates exhaustion-associated proteins in T cells, highlighting its potential to enhance T cell functionality. Specifically, CleTAC-mediated degradation promotes T cell proliferation, sustains activation, and improves antitumor efficacy of CAR-T therapies, particularly against solid tumours. Another significant advantage of the CleTAC approach is its simplicity and safety profile. By incorporating the CleTAC plasmid with CAR constructs, functional enhancement can be achieved with one transfection. The compact design of the CleTAC plasmid streamlines manufacturing and minimizes off-target effects, making it a promising adjunct to current immunotherapeutic platforms.

However, our findings also reveal a significant challenge: CAR-T cell meditated trogocytosis (CMT) induces T cell cannibalism and dysfunction, thereby compromising antitumor efficacy 51,52. To mitigate this effect, we deployed CleTAC to target HER2 on the CAR-T cell surface, directing it for lysosomal degradation. CleTAC-mediated HER2 clearance markedly reduced CMT effects, illustrating its unique capacity to eliminate cell-surface antigens at the protein level—an outcome that cannot be achieved through DNA- or RNA-based methods. Thus, CleTAC represents a promising strategy to overcome CMT-associated dysfunction and enhance the therapeutic performance of CAR-T cells.

We compared CleTAC side by side with three established strategies: PROTAC, TransTAC, and CAR T combined with the anti CTLA 4 antibody ipilimumab. The rationale for selecting TransTAC over LYTAC lies in the fact that both utilize the lysosomal pathway for degradation, but TransTAC specifically targets membrane proteins, making it a more appropriate comparator to CleTAC, which also targets membrane proteins. Our results show that CleTAC surpasses both the anti CTLA 4 antibody and PROTAC methods in antitumor efficacy, whereas TransTAC shows a modest improvement over CleTAC. These findings underscore CleTAC's unique strength in directly degrading membrane bound targets while also identifying opportunities for further optimisation to close the remaining gap with TransTAC.

While these results are encouraging, several key issues must be addressed before CleTAC can advance toward clinical application. First, our *in vitro* findings need to be validated in relevant animal models to assess tissue distribution, systemic toxicity, and long-term pharmacodynamics under physiological conditions. Secondly, further refinement of the CleTAC design is required to improve target specificity and minimize off-target degradation, a challenge similarly encountered in PROTAC systems 71. Finally, the current range of well-characterized, multivalent protein-interaction modules is limited, restricting the range of proteins that can be targeted by CleTAC systems 72. Addressing these challenges will be essential for the successful translation of CleTAC into a therapeutic platform.

Nevertheless, ongoing scientific and technological advancements, coupled with the integration of multidisciplinary theoretical frameworks, promise to significantly expand CleTAC's applicability across diverse therapeutic and research domains.

## Methods

### Institutional approval

All animal experiments were conducted under the supervision of the Nanchang University Animal Centre and in compliance with approved ethical guidelines (project approval number: NCULAE-20240326001). Human sample analyses were performed in accordance with an approved protocol at The Second Affiliated Hospital of Nanchang University (project approval number: IIT-O-2024-013).

### Plasmid construction

Lentiviral constructs were generated by cloning target coding sequences into the lpCDH-puro-myr-HA-Akt1 plasmid backbone (Addgene #46969). Inserted fragments were commercially synthesized (GENEWIZ) and integrated into the lentiviral transfer vector via Gibson Assembly. Control groups were prepared by removing the YVKM fragment through plasmid linearization, whereas FVKM variants were generated using PCR amplification with primers designed for point mutations. Subsequent generations of CleTAC plasmids followed similar procedures. Transient transfections employed Lipofectamine 3000 (Invitrogen, L3000015), strictly adhering to the manufacturer's protocol. We consistently used 24 h and 3 μg of CleTAC in subsequent transfection experiments.

### Cell culture

293T and U251 cell lines (ATCC-derived) were propagated in DMEM (Sigma) containing 10% heat-inactivated fetal bovine serum (FBS, Gibco) plus 100U/ml penicillin and 100µg/ml streptomycin (Gibco). CAR-T cells were differentiated from healthy donor-derived peripheral blood mononuclear cells (PBMCs) cultured in X-VIVO 15 mediums (Gibco) supplemented with 5% human AB serum (Gibco) and 10ng/ml IL-2 recombinant protein (Peprotech, #200-02). All cell types were incubated at 37 °C under 5% CO₂ with controlled humidity.

### T cell expansion

HER2-expressing irradiated NIH/3T3 feeder cells (3×10⁵/well) were co-cultured with CAR-T cells (1×10⁶/ml) in cytokine-free RPMI-1640 medium plus 10% FBS. CAR-T populations were enriched via magnetic bead isolation four days post-transduction, using CD271 (LNGFR)-PE antibody (Miltenyi Biotec, #130-113-421) and anti-PE microbeads (Miltenyi Biotec, #130-048-801). Functional assays and cell quantifications were conducted seven days post-stimulation using an automated cell counter (Thermo Fisher).

### Lentivirus production

293T cells (4×10⁷) were seeded onto 10 cm dishes and transfected with 20 µg transfer plasmid, 10 µg psPAX2 (Addgene, #12260), and 5 µg pMD2.G (Addgene, #12259) using 87.5 µl LipoD293. Supernatants were collected and subpackaged after 48 h, aliquoted, and stored at -80 °C. Lentiviral particles were concentrated with Lenti-X Concentrator (Clontech, #631231) prior to transduction of 293T, U251, and primary T cells for 24-48 h.

### Human T cell phenotyping

T cell surface markers were assessed using antibodies specific for IL-2 receptor alpha (CD25, Abcam, ab25534), CD45 (Alexa Fluor 488, Abcam, ab2200315), and CCR7 (APC, Abcam, ab155382). Transfected T cells underwent sorting, expansion, and a 10-day resting period prior to analysis. Cells were stained in PBS buffer containing 2% FBS at 4 °C for 15 min, washed, and analysed by flow cytometry.

### Generation of stable cell lines

Stable cell lines were established via lentiviral transduction, followed by enhanced green fluorescent protein (EGFP) reporter-based purification through fluorescence-activated cell sorting (FACS).

### Flow cytometry

For EGFP intensity assessment, cells were dissociated using EDTA-free trypsin, washed, resuspended in PBS, and immediately analysed via CytoFLEX flow cytometer (Beckman Coulter). A minimum of 1×10⁴ cells per sample were analysed, and data were processed with FlowJo V10 software (TreeStar Inc).

### Reverse transcription quantitative PCR (RT-qPCR) analysis

Total RNA was extracted with the Super FastPure Cell RNA Isolation Kit (Vazyme, #RC102) and quantified using a NanoDrop spectrophotometer (Thermo Scientific). cDNA synthesis from 0.4-1 µg RNA utilized SuperScript IV reverse transcriptase (Thermo Fisher, #18090050). qPCR was conducted with PowerTrack SYBR Green Master Mix (Thermo Fisher, #A46012), with gene expression normalized to GAPDH via the ΔΔCt method.

### Design and construction of the inner membrane segment binding protein

Target-binding proteins were designed using RFdiffusion in combination with ProteinMPNN and AlphaFold 3. Dimeric structures of HER2 and binding proteins were predicted with AlphaFold 3. Three configurations demonstrating high computational scores were selected for experimental validation, adhering primarily to default optimization parameters specified by RFdiffusion.

### Immunoprecipitation (IP)

Cell samples were collected and lysed in EBC buffer (120 mM NaCl, 50 mM Tris, pH 7.5, 0.5% NP-40), supplemented with protease inhibitors (Bimake, #B14002) and phosphatase inhibitors (Bimake, #B15002). Protein concentrations were quantified using the BCA assay on a Multiskan FC microplate reader (Thermo Scientific, #51119180ET). For immunoprecipitation assays, 1-2 mg of protein lysates were incubated overnight at 4 °C with anti-Flag or anti-HA antibodies conjugated to beads. Immunocomplexes were subsequently washed four times with NETN buffer (100 mM NaCl, 20 mM Tris, pH 8.0, 1 mM EDTA, 0.5% NP-40). Both lysate proteins and immunoprecipitated proteins were resolved by SDS-PAGE, transferred to membranes, and detected using specific antibodies via enhanced chemiluminescence (ECL).

### Immunofluorescence (IF)

293T cells (200 μL of complete DMEM medium per well) were cultured on cover glasses in 12-well plates (WHB Scientific, #WHB-12-CS) for 24 h before transfection. Complexes composed of 0.23 μL Xfect transfection reagent and 750 ng plasmid DNA were prepared in 10 μL Xfect reaction buffer and incubated for 10 min. Following media replacement with 130 μL fresh complete DMEM, the prepared complexes were added to each well. After incubation for 24 h at 37 °C, cells were gently washed with cold PBS and subsequently fixed and permeabilized using the BD Fixation/Permeabilization Kit (BD Biosciences, #554714). Samples were blocked for 30 min in PBST buffer (PBS containing 0.1% Tween-20) supplemented with 0.3 M glycine and 1% BSA. Primary antibodies diluted in blocking solution were applied overnight at 4 °C. After thorough washing with PBS, secondary antibodies were incubated for 1 h at room temperature in darkness. Samples were rinsed again with PBS prior to imaging. Fluorescence imaging was performed using a Nikon Ti confocal microscope equipped with a Yokogawa CSU22 spinning disk module, with sequential excitation at wavelengths of 405 nm, 488 nm, 561 nm, and 647 nm. Colocalization analyses were conducted by calculating Pearson's correlation coefficients using NIS-Elements AR software (Nikon).

### Cytolysis assay

T cell cytotoxicity was measured via bioluminescent assay using luciferase-expressing U251 cells. Effector T cells were co-incubated at specific E:T ratios. Luminescence was quantified using a PerkinElmer plate reader, and cytotoxicity calculated as specific lysis percentage:

Specific Lysis (%) = [1-RLU (co-culture) / RLU (target cells alone)] × 100%

### ELISA assay

Following the manufacturer's protocols, levels of IL-2, IL-6, IL-1β, and IFN-γ in co-culture supernatants were analyzed via ELISA (Thermo Fisher), that cultured supernatants (100μl) were harvested after defined incubation periods for cytokine assessment.

Serum isolated from mouse blood was used to determine alanine transaminase (ALT), aspartate transaminase (AST) and blood urea nitrogen (BUN) activities by Amplex Red ALT Activity Assay Kit, Amplex Red AST Activity Assay Kit and Urea Nitrogen Assay Kit (Beyotime, China) according to manufacturer's protocol. The OD values were measured by an enzyme-labeled instrument at A570. The concentration of ALT, AST and BUN in the sample was calculated by the standard curve.

### CCK-8 assays

To systematically assess the impact of CleTAC treatment on cell proliferation, CCK-8 assays were conducted using the Cell Counting Kit-8 (Solarbio, China) according to the manufacturer's standardized protocol. CAR-T cells were plated in a 96-well plate at a density of 2,000 cells per well in triplicates. Following a 12 h pre-incubation period to allow cell adhesion and stabilization, 10 μL of CCK-8 solution was added to each well at four designated time points post-treatment (0, 24, 48, and 72 h). After a 1 h incubation at 37 °C, cell proliferation was quantified by measuring optical density (OD) at 450 nm using a microplate reader.

### *In vivo* mouse studies

An *in-situ* tumor-bearing mouse model was established using C57BL/6 nude mice. The 251-tumor cell line (5×10⁵ cells) was stereotactically implanted into the mouse brain at coordinates 4 mm posterior and 1 mm lateral to the anterior fontanelle. CAR-T cells were intravenously administered on days 0 and 7 post-implantation. Tumor progression was monitored using *in vivo* imaging techniques. Mouse body weight measurements were recorded every three days. Tumor growth dynamics were assessed through *in vivo* imaging and histological examination via haematoxylin-eosin (HE) staining. By evaluating survival rates and tumor development, the enhanced antitumor efficacy of CAR-T cells modified by CleTACs was comprehensively analysed. All the animal experiments in this study involved 3 mice in each group for repetition.

### *In vivo* bioluminescence imaging

D-Luciferin potassium salt was injected intraperitoneally into mice prior to bioluminescence signal acquisition using an IVIS Spectrum system (Perkin Elmer). Data analysis employed Living Image software (v4.3.1).

### Analyses of serum cytokines

Mouse serum cytokine levels (IL-1β, IL-2, IL-6, IFN-γ) were quantified using commercial ELISA kits (Thermo Fisher).

### Statistical analysis

Data were analysed with GraphPad Prism 6 using two-way ANOVA with Bonferroni correction. Results represent means from three independent experiments, with significance thresholds defined as ns (p>0.05), * (p≤0.05), *** (p≤0.001), and **** (p≤0.0001).

## Supplementary Material

Supplementary figures and table.
