# Supplementary material for: Clathrin mediated endocytosis targeting chimera for targeted membrane proteins degradation and enhance CAR-T cell anti-tumor therapy
Source: Theranostics. 2026 Jan 1;16(1):254–71. doi: 10.7150/thno.114005 (PMC12665105; doi:10.7150/thno.114005)
Supplement: Supplementary file 1 — Supplementary figures and table. [file thnov16p0254s1.pdf]

Table S1: Sequence information of single gRNA targeting in the designed CRISPR/Cas9 knockout system.

Gene	***AP1M1***	***AP2M1***	***AP3M1***
TargetSeq1	CTTACGTACCTGGATGCCGG	ATCAGCTTTATCCCGCCAGA	TTTACCCCTGCCCGACGCCA
TargetSeq2	ACGGGGGGGTCCGTTTCATG	CTATAAGATGTGTGACGTGA	TATGTTGGACAGCTGCCCGG
TargetSeq3	ATCTCGAACTTGACACTGAT	CCGCTTAACGTGGAAGAAGC	GATGCTTGCATTAAACTATC
TargetSeq4	CGACACGCACGCGTTCTTCT	TACCCACAGAATTCCGAGAC	CAGATTCCCAACGCTTGAAC

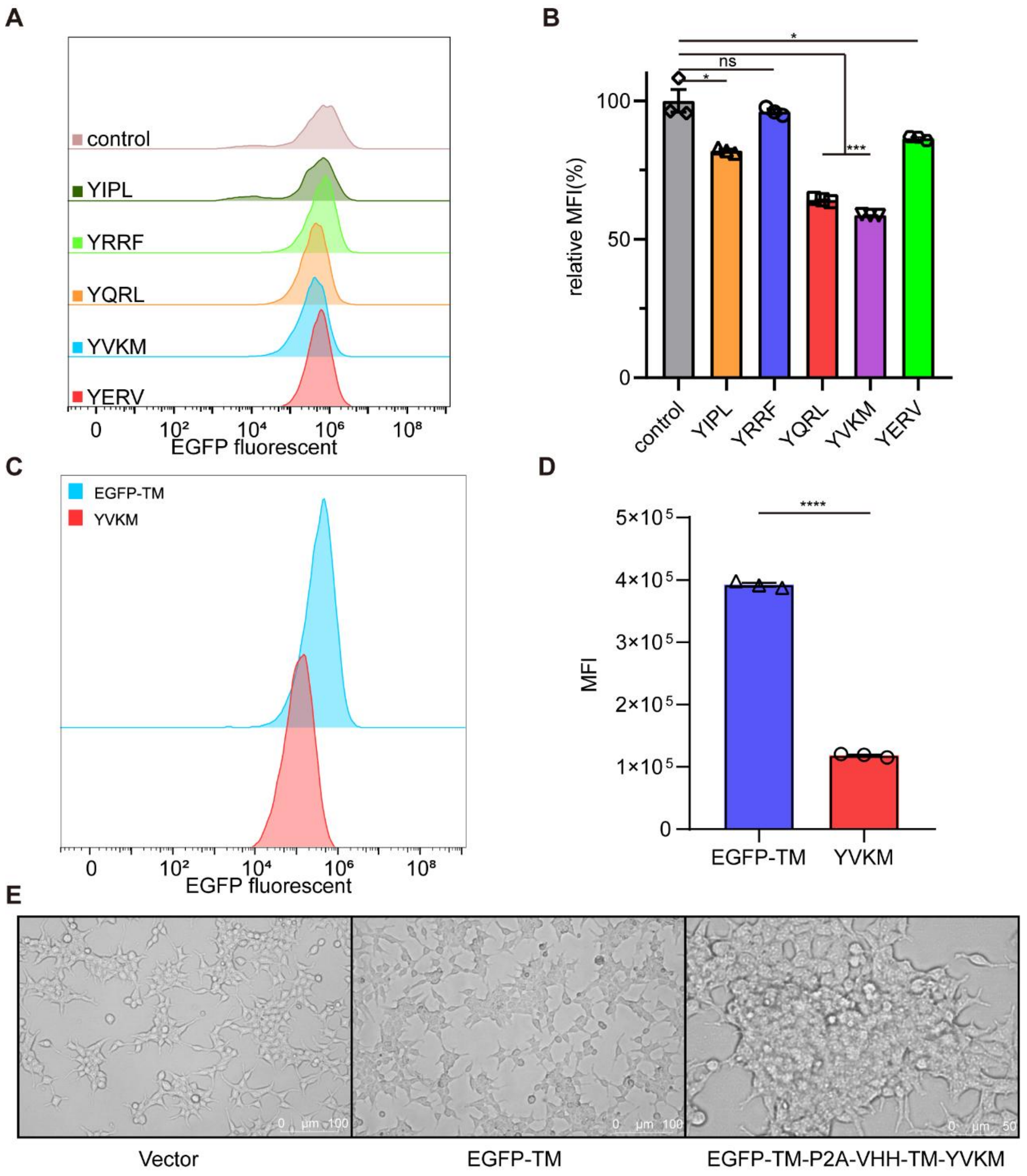

Figure S1: Validation of degradation efficiency of YXXΦ motifs.

(A) Distribution of EGFP fluorescent cells in 293T cells stably expressing different YXXΦ motifs. CTLA4 (YVKM, 201-204), HCV (YIPL, 136-139), m04/gp34 (YRRF, 247-250), TGN38 (YQRL, 430-433) and EREG (YERV, 156-159) (B) The MFI exhibited by 5 motifs after intracellular expression was displayed. (C) Distribution of EGFP fluorescent cells in 293T cells stably expressing VHH-TM-YVKM. (D) The

relative mean fluorescence intensity (MFI) exhibited by EGFP-TM and VHH-TM-YVKM after intracellular expression was displayed. (E) Cell image of VHH-TM-YVKM transfected cells under light microscopy.

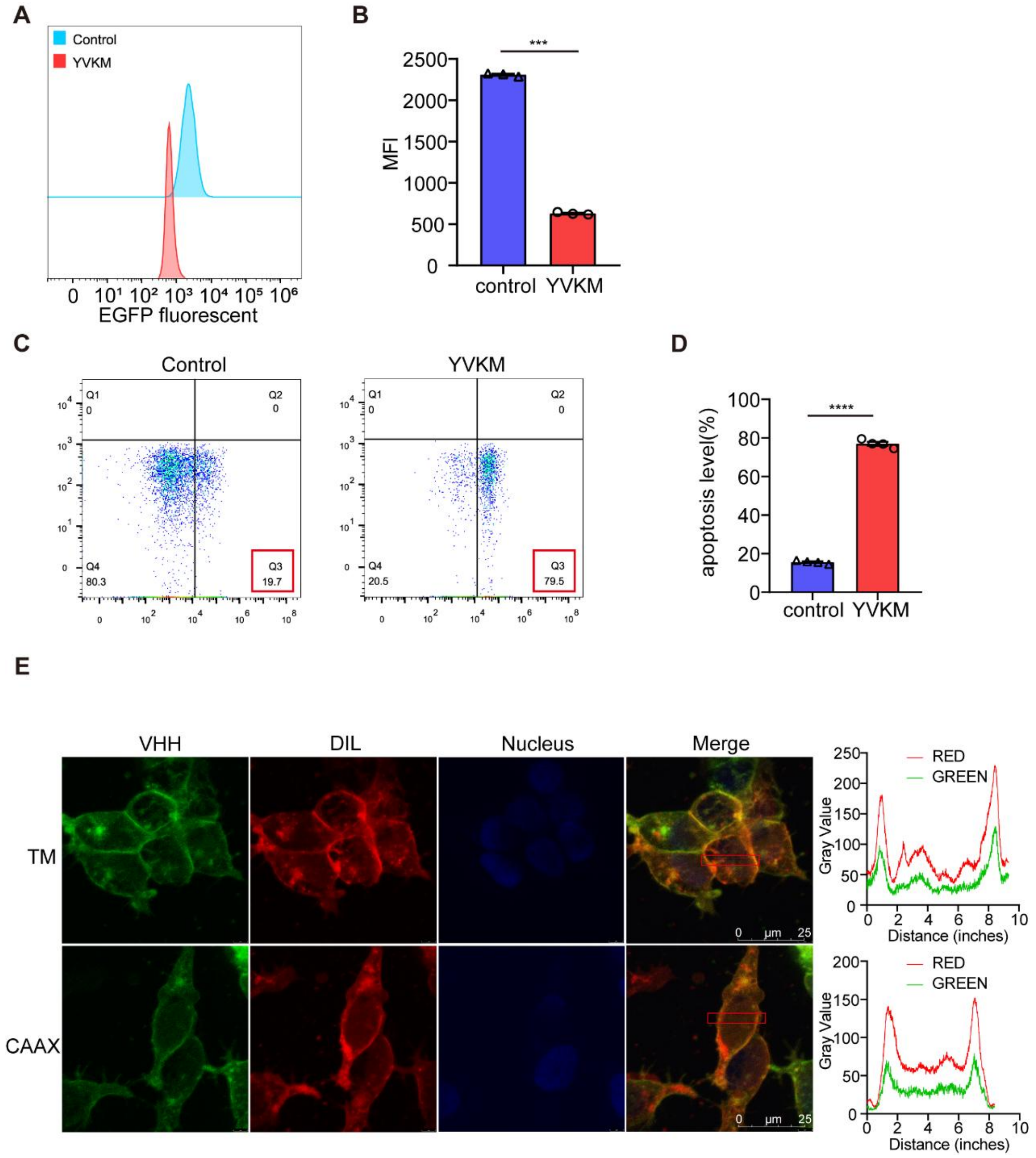

Figure S2: VHH-TM-YVKM can effectively reduce the surface EGFP of CAR-T cells without fratricide.

(A) Distribution of EGFP fluorescent cells in human T cells stably expressing VHH-TM-YVKM. (B) The relative mean fluorescence intensity (MFI) exhibited by VHH-

TM-YVKM after intracellular expression was displayed in T cells. (C) Apoptotic rates of T cells transfected by VHH-TM-YVKM for 24h were tested by flow cytometry. (D) Percentage of apoptotic levels of T cells transfected by VHH-TM-YVKM for 24h, n=4, data are shown as mean ± SEM. (E) Representative images for the cellular localization of CleTAC degrader and DIL orange red cell membrane fluorescent dye in live 293T cells. Scale bar: 20 μm.

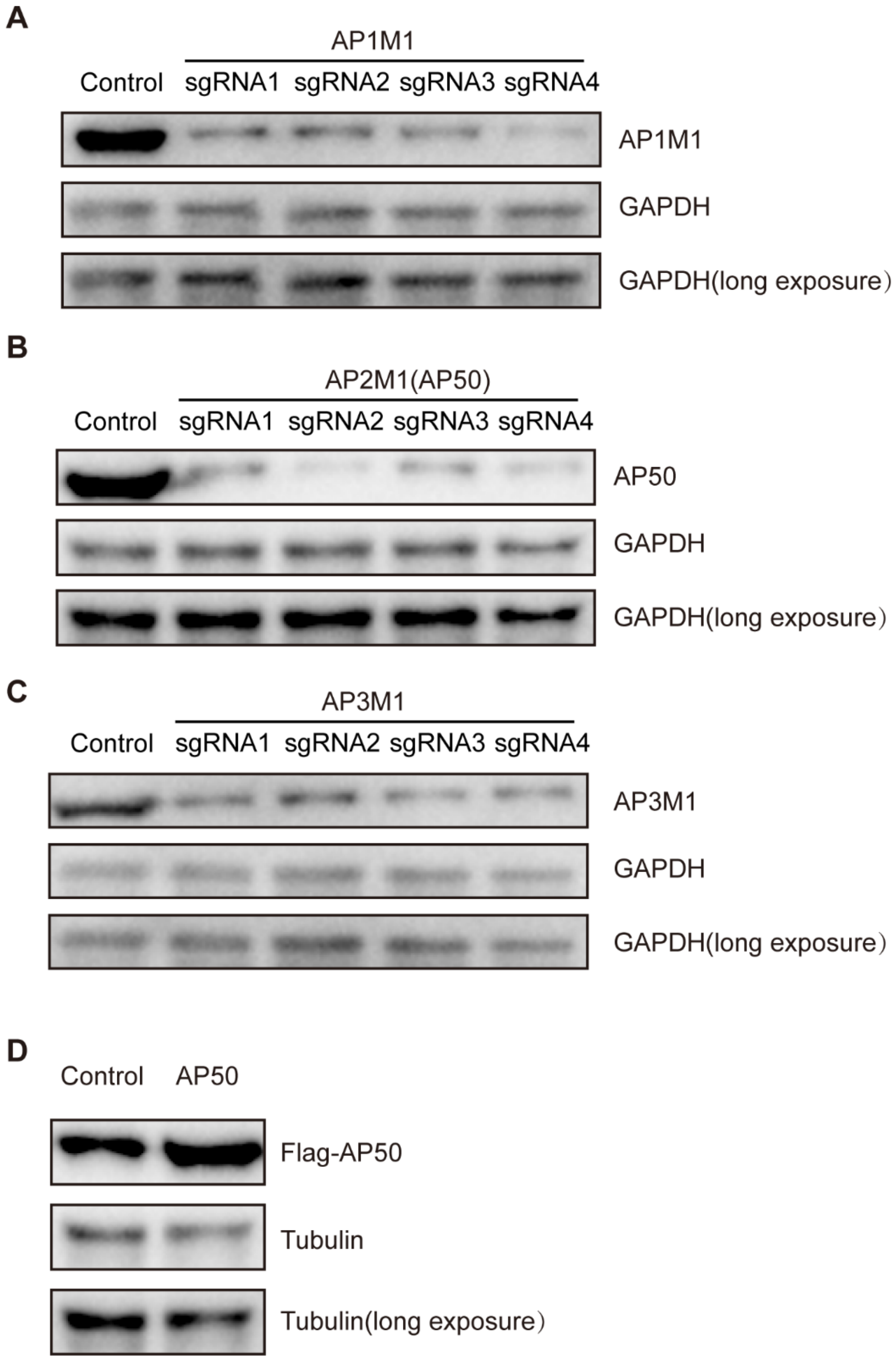

Figure S3: Western blot validation of knockout and overexpression plasmids.

(A-C) Western blot analysis of AP1M1 (A), AP2M1 (B) and AP3M1 (C) levels in knockout 293T cells by different sgRNAs. (D) Western blot analysis of AP50 levels in 293T cells which overexpress Flag-AP50 plasmid.

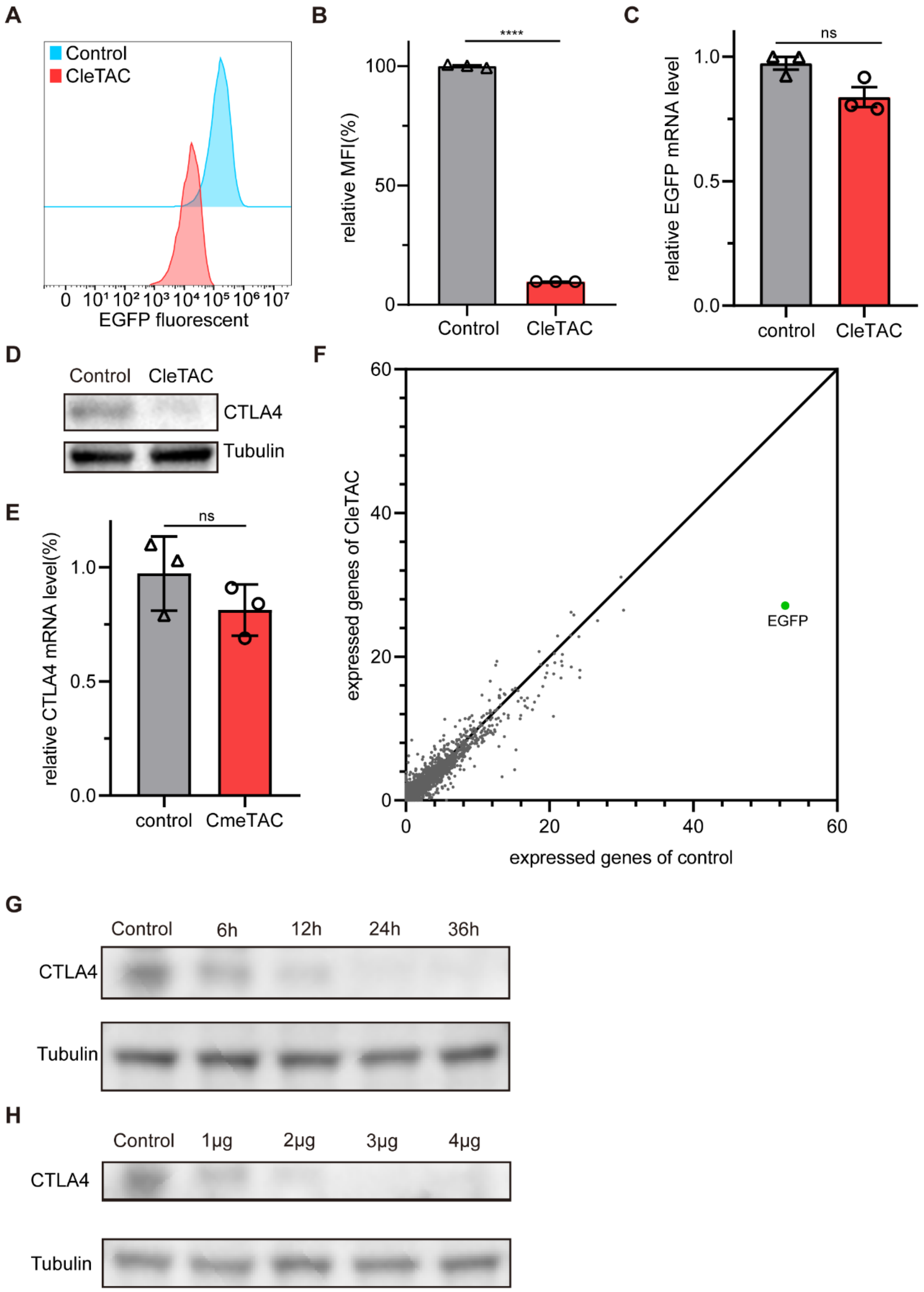

Figure S4: Flow cytometry and RT-qPCR show that CleTACs degrade target protein through post-transcriptional mechanism.

(A, B) Distribution of EGFP fluorescent cells (A) and relative mean fluorescence

intensity (B) in 293T cells stably expressing CleTAC. (C) Relative EGFP mRNA levels of 293T cells stably expressing CleTAC. (D) Western blot analysis of CTLA4 level in CleTAC CAR-T cells compared with control group CAR-T cells. (E) Relative CTLA4 mRNA levels of CAR-T cells stably expressing CleTAC. (F) Volcano plot. The log2 FC indicates the mean expression level for each gene. Each dot represents one gene. Black dots represent no significant DEGs between CleTAC group and control group, the green dots represent down-regulated genes and red dots represent up-regulated genes. (G-H) Western blot analysis of CTLA4 level in different transfection times (G) and different transfection concentrations (H) CleTAC plasmid compared with control group.

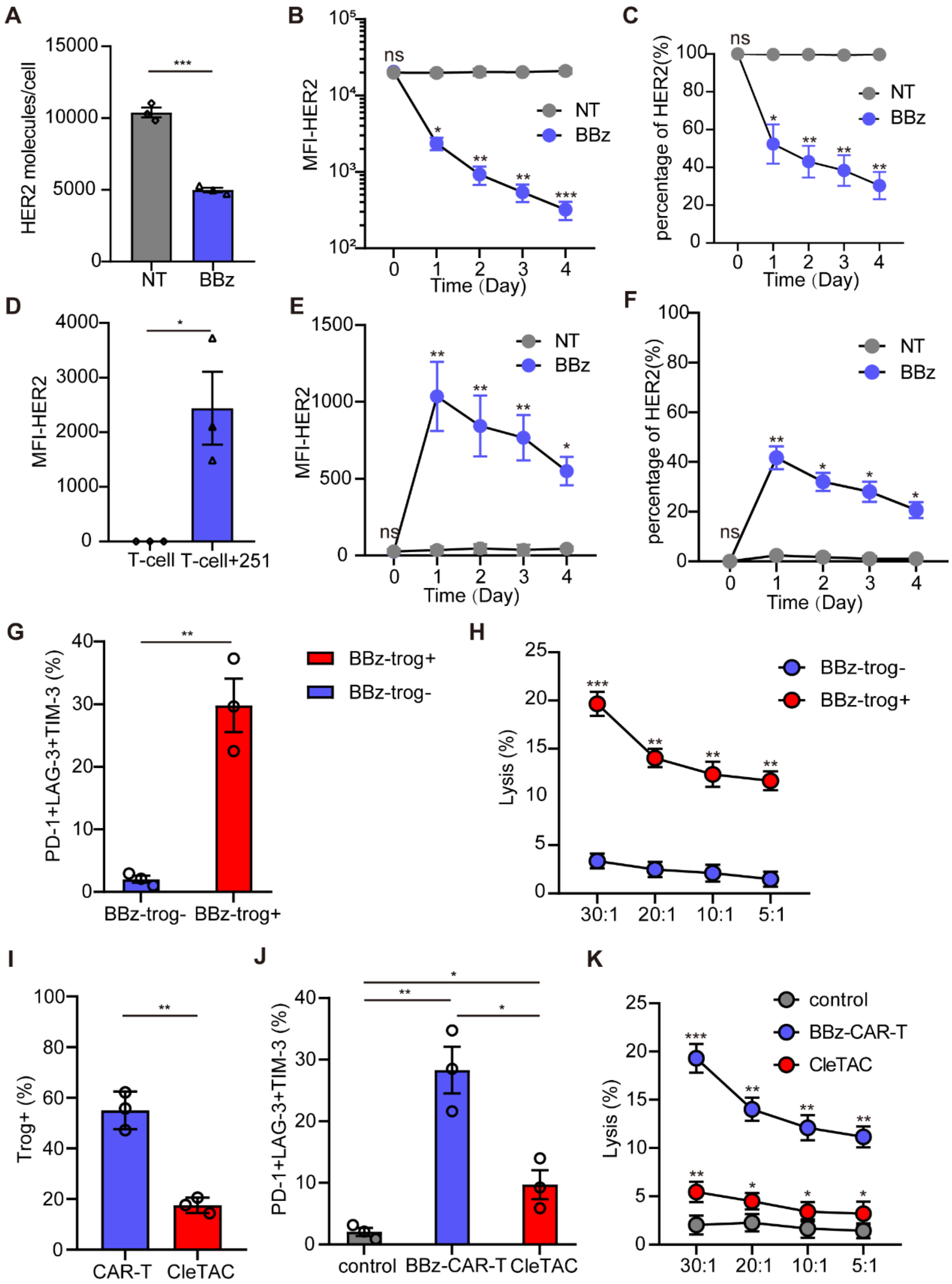

Figure S5: The CleTAC-mediated HER2 degradation can mitigate CAR-mediated trogocytosis and enhance CAR-T cell efficacy.

(A) HER2 expression in 251 cells treated with BBζ CAR-T cells (n=3). (B-C) Mean fluorescence intensity (MFI) and percentage of HER2 on 251 cells (n=3). (D-F) MFI

(D, E) and percentage (E) of HER2 on CAR-T cell surface. n=3, data are shown as mean ± SEM. (G) Percentage of CAR-T cells co-expressing PD-1, LAG-3 and TIM-3 after stimulated by 251 cells (n=3). (H) Luciferase-based cytotoxic assay using 251 target cells at several E: T ratios (n=3 donors). (I) HER2+ percentage of CAR-T cells stably expressing HER2-CleTAC (n=3). (J) Percentage of HER2-CleTAC CAR-T cells co-expressing PD-1, LAG-3 and TIM-3 (n=3). (K) Luciferase-based cytotoxic assay of HER2-CleTAC CAR-T cells using 251 target cells at several E: T ratios (n=3).

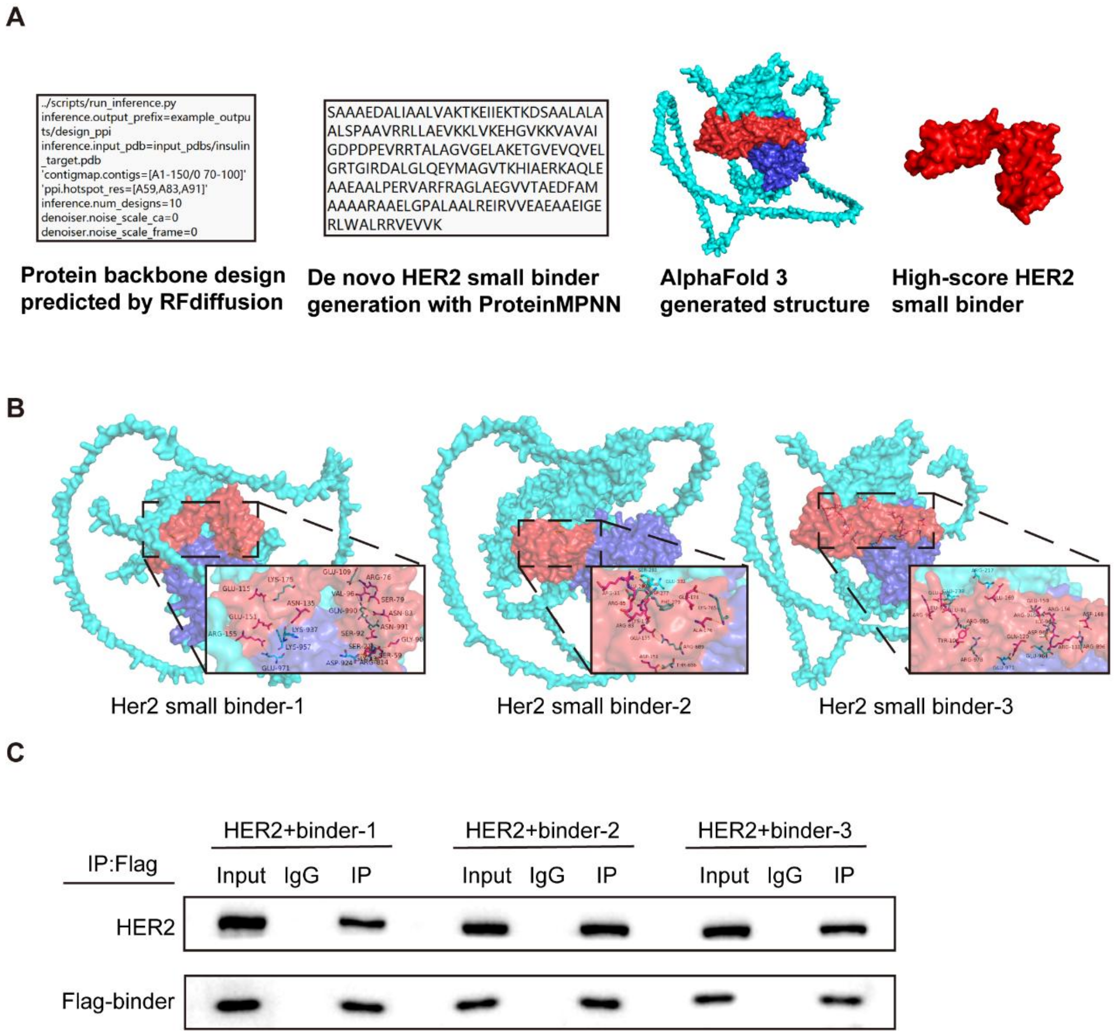

Figure S6: The design and validation of HER2-binding proteins.

(A) Scheme of HER2 small binder generation. (B) Predicted structure of dimeric interface of HER2 and small binders by AlphaFold 3. Small binders are colored in red and intracellular segment of HER2 in colored in blue. (C) Co-immunoprecipitation (co-IP) of HER2 from 293T cells after transfection of plasmids that encode HER2 binders. HER2 binders were immunoprecipitated via the Flag-tag; binder-bound protein complexes were then captured from the lysates and blotted for the HER2 and FLAG-tag (HER2 binders).

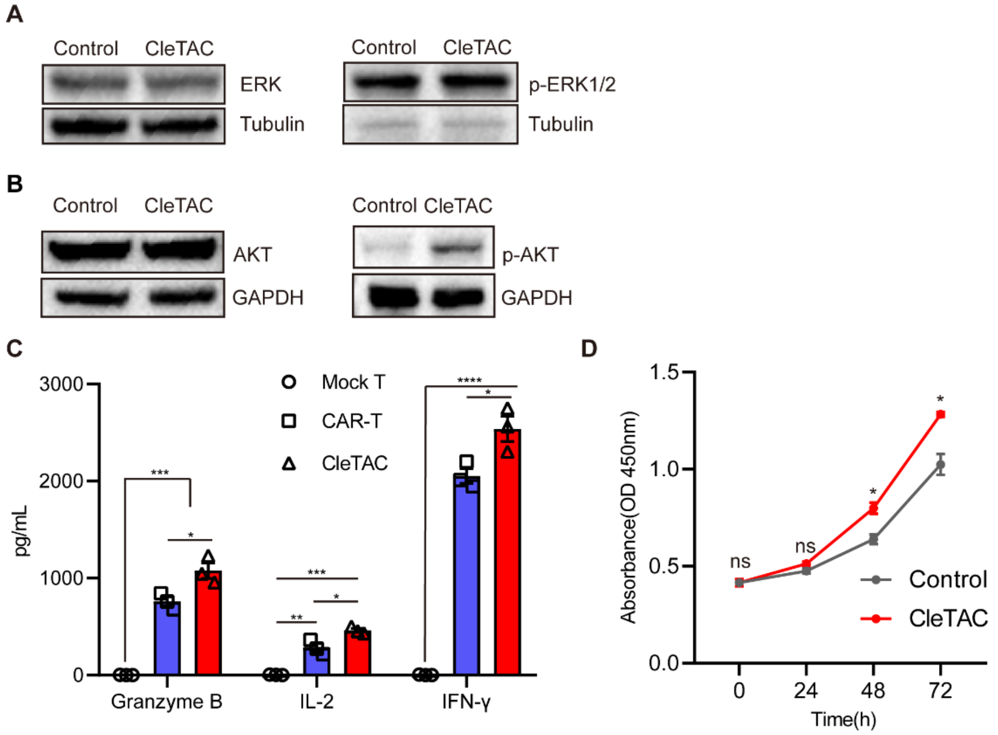

Figure S7: Effect of CleTAC-mediated CTLA4 degradation on CAR-T cell signaling and activity.

(A) Western blot analysis of ERK and p-ERK levels in CleTAC CAR-T cells compared with control group CAR-T cells. (B) Western blot analysis of AKT and p-AKT levels in CleTAC CAR-T cells compared with control group CAR-T cells. (C) Quantification of gramzyme B, IL-2 and IFN-γ expression in different treatment T

cells (n=3). (D) Cell viability measured by cell counting kit-8 of CleTAC CAR-T cells. n=3. For all bar plots, data are shown as mean ± SEM.

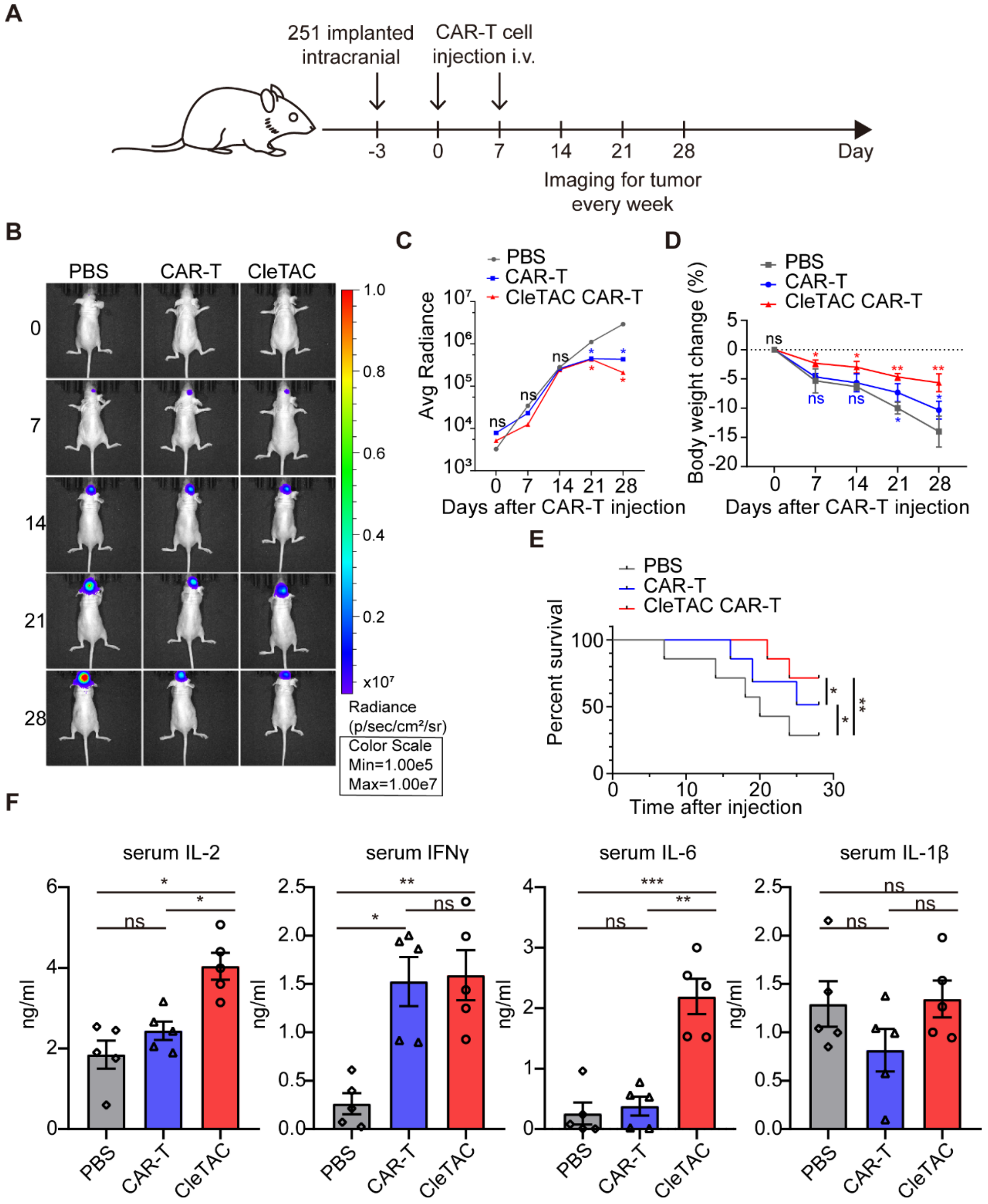

Figure S8: Validation of reduced I.V. dose CleTAC CAR-T cells therapy *in vivo*. (A) Flowchart for intracranial construction of the U251 model and subsequent treatment. (B) Bioluminescence imaging of nude mice xenografted with luciferase-

infected U251 tumor cells. From left to right shows PBS, CAR-T and CleTAC CAR-T treatment. (C, D) Average radiance of bioluminescence (C) and percentage of body weight change (D) for different treatment groups of nude mice. (E) Monitoring the survival status of mice after T cell injection. (F) Quantification of serum IL-2 (n=5), IFN-γ (n=5) IL-6 (n=5) and IL-1β (n=5) expression. For all bar plots, data are shown as mean ± SEM.

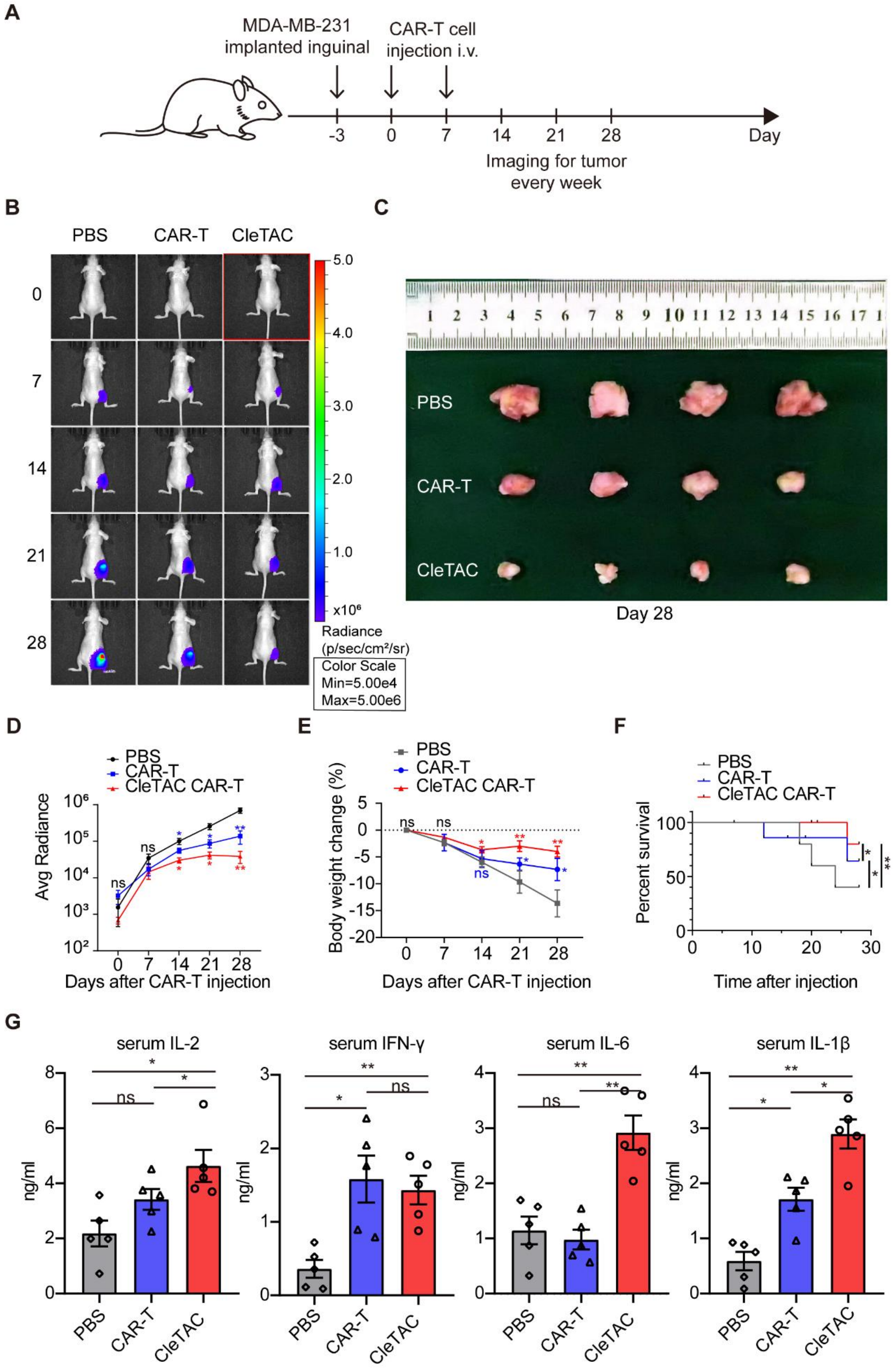

Figure S9: Validation of CleTAC CAR-T cells therapy in breast carcinoma xenograft

model *in vivo*.

(A) Flowchart for inguinal construction of the MDA-MB-231 model and subsequent treatment. (B) Bioluminescence imaging of nude mice xenografted with luciferase-infected MDA-MB-231 tumor cells. From left to right shows PBS, CAR-T and CleTAC CAR-T treatment. (C) Image of isolated flanking tumors. From top to bottom shows PBS, CAR-T and CleTAC CAR-T treatment. (D, E) Average radiance of bioluminescence (D) and percentage of body weight change (E) for different treatment groups of nude mice. (F) Monitoring the survival status of mice after T cell injection. (G) Quantification of serum IL-2 (n=5), IFN-γ (n=5) IL-6 (n=5) and IL-1β (n=5) expression. For all bar plots, data are shown as mean ± SEM.
